# Innovative optimization for enhancing Pb^2+^ biosorption from aqueous solutions using *Bacillus subtilis*

**DOI:** 10.3389/fmicb.2024.1384639

**Published:** 2024-08-08

**Authors:** Reyad M. El-Sharkawy, Mohamed Khairy, Mohamed H. H. Abbas, Magdi E. A. Zaki, Abdalla E. El-Hadary

**Affiliations:** ^1^Botany and Microbiology Department, Faculty of Science, Benha University, Benha, Egypt; ^2^Chemistry Department, College of Science, Imam Mohammad Ibn Saud Islamic University (IMSIU), Riyadh, Saudi Arabia; ^3^Chemistry Department, Faculty of Science, Benha University, Benha, Egypt; ^4^Soils and Water Department, Faculty of Agriculture, Benha University, Benha, Egypt; ^5^Biochemistry Department, Faculty of Agriculture, Benha University, Benha, Egypt

**Keywords:** *Bacillus subtilis*, optimization, biosorption, lead, low-cost remediation, definitive screening design, artificial neural network

## Abstract

**Introduction:**

Toxic heavy metal pollution has been considered a major ecosystem pollution source. Unceasing or rare performance of Pb^2+^ to the surrounding environment causes damage to the kidney, nervous, and liver systems. Microbial remediation has acquired prominence in recent decades due to its high efficiency, environment-friendliness, and cost-effectiveness.

**Methods:**

The lead biosorption by *Bacillus subtilis* was optimized by two successive paradigms, namely, a definitive screening design (DSD) and an artificial neural network (ANN), to maximize the sorption process.

**Results:**

Five physicochemical variables showed a significant influence (*p* < 0.05) on the Pb^2+^ biosorption with optimal levels of pH 6.1, temperature 30°C, glucose 1.5%, yeast extract 1.7%, and MgSO_4_.7H_2_O 0.2, resulting in a 96.12% removal rate. The Pb^2+^ biosorption mechanism using *B. subtilis* biomass was investigated by performing several analyses before and after Pb^2+^ biosorption. The maximum Pb^2+^ biosorption capacity of *B. subtilis* was 61.8 mg/g at a 0.3 g biosorbent dose, pH 6.0, temperature 30°C, and contact time 60 min. Langmuir’s isotherm and pseudo-second-order model with R^2^ of 0.991 and 0.999 were suitable for the biosorption data, predicting a monolayer adsorption and chemisorption mechanism, respectively.

**Discussion:**

The outcome of the present research seems to be a first attempt to apply intelligence paradigms in the optimization of low-cost Pb^2+^ biosorption using *B. subtilis* biomass, justifying their promising application for enhancing the removal efficiency of heavy metal ions using biosorbents from contaminated aqueous systems.

## 1 Introduction

Water contamination caused by heavy metal ions has been considered one of the most universal environmental hazards and health risks in developed and developing countries. However, it is continuously growing because of the discharge of improperly or incompletely treated industrial effluents in the aquatic environment due to urbanization and industrialization (Arsenie et al., 2022; Nguyen et al., 2022; Ahn et al., 2023; Kang et al., 2023; Li et al., 2023; Zinicovscaia et al., 2023; El-Beltagi et al., 2024). Certain heavy metals in trace amounts, including Zn^2+^, Cu^2+^, and Fe^2+^, are essential for the different biological activities of organisms. However, heavy metals at higher levels may be highly toxic and inhibit different metabolic activities in living organisms (Badawi et al., 2021; Oliveira et al., 2023; Yu et al., 2023; El-Sharkawy et al., 2024). The discharge of heavy metals, in free forms, in water bodies, farmland soil, urban soil, and even the atmosphere could be prolonged to the animal and human food chains, thereby critically threatening the health and activities of different living organisms (El-Naggar et al., 2020; Hu et al., 2020; Arce-Inga et al., 2022; Kang et al., 2023; Racić et al., 2023; Shehnaz et al., 2023). Among different polluting metal ions, Pb^2+^ and Hg^2+^ have certain industrial significance, particularly in producing batteries, paints, and alloys, which makes them still employed on a huge scale. The industrial effluents of such industrial activities are suggested to contain high concentrations of heavy metal ions that can rapidly accumulate and contaminate the surrounding environment. Therefore, eradicating various heavy metal ions from the terrestrial and aquatic environment is highly desirable (Arsenie et al., 2022; Ahn et al., 2023; Shaaban Emara et al., 2023).

Lead (Pb^2+^) is considered a substantial and ubiquitous heavy metal that causes risky living organisms’ health issues and environmental pollution (Sharma et al., 2022; Racić et al., 2023). Lead is released through different industries, including pigments, mining, batteries, construction, Pb^2+^-containing insecticides, and water pipes. The discharge of lead, even at low concentrations, into the surrounding ecosystems may lead to various hazard issues to the soil, plants, animals, microbes, and particularly to human health, like damage to the liver, kidneys, nervous system, and growth (Çolak et al., 2011; Arce-Inga et al., 2022; Hidangmayum et al., 2022; Shaaban Emara et al., 2023). The concentration of Pb^2+^ should be within the permissible low limit of 0.1 mg/L for polluted water and 0.05 mg/L for drinking water based on the World Health Organization (WHO) guidelines. However, it is a great challenge to reduce the concentration of Pb^2+^ to the allowed safe level (Alzahrani et al., 2022; Chintalapudi et al., 2022; Karn et al., 2023).

Industrial effluents can be treated using various available remediation strategies to reduce the concentration of contaminating species to a permissible level. In the most general sense, chemical, physical, and microbial methods can be employed for the remediation of heavy metals. However, the remediation of heavy metal contaminants is difficult due to their non-biodegradable properties (El-Shora and El-Sharkawy, 2020a,b; El-Sharkawy et al., 2022; El-Shora et al., 2022; Wu et al., 2022; El-Sharkawy and Abbas, 2023; Liaqat et al., 2023). The physic-chemical methods, including precipitation, membrane technology, and activated carbon sorption, have several disadvantages that reduce their applicability, such as high cost, partial metal ion remediation, inadequate selectivity, recontamination by secondary metabolite byproducts, and non-adequacy at high and minute metal ion concentrations (Wang et al., 2010; Hidangmayum et al., 2022; Palmieri et al., 2022; Li et al., 2023; Liaqat et al., 2023).

In contrast, microbial methods are promising unconventional strategies to remediate heavy metals from polluted environments due to their fast, simple, easy adaptation to various operating conditions, cost-effectiveness on a large-scale operation, cleanliness, biosorption ability of various contaminating species, and eco-friendly techniques when compared with traditional physicochemical methods (Venil et al., 2011; Pan et al., 2017; Yin et al., 2019; Sharma et al., 2021, 2022). The broad applicability of microbial sorption processes in the treatment of industrial effluents is quite limited owing to various limitations, like the selection of low-cost and suitable biosorbents with enough quantity to ensure accessibility to industrial wastewater and the continuity of the bioremediation process, particularly for large-scale applications (El-Naggar et al., 2020; Baran et al., 2022; Shaaban Emara et al., 2023).

Bacteria are highly efficient for toxic heavy metal ion remediation when compared with fungi, actinomycetes, and other biosorbents, particularly in solutions with low concentrations of heavy metals. This may be attributed to their minor generation time, small size, resistance mechanism, and adaptability to various environments of biosorbent bacteria. The lipid and protein content of the bacterial surface shows different functional groups that confer binding sites for metal ion biosorption (El-Naggar et al., 2020; El-Sayed et al., 2021; Ahn et al., 2023; El-Sharkawy and Abbas, 2023; Krucoń et al., 2023; Li et al., 2023).

Metal ion remediation using different types of microorganisms can be performed by various strategies such as cellular mechanisms, cation-diffusion transport, ABC transport, ATPase transport, and morphological alterations of microbial cells, as reported by Pan et al. (2007), El-Sayed et al. (2021), El-Shora et al. (2021), Sharma and Shukla (2021), El-Shora et al. (2022), Hidangmayum et al. (2022), Ahn et al. (2023), and Upadhyay et al. (2023). Bioaccumulation and biosorption are other methods used by microbial cells to eliminate heavy metal ions. Biosorption is considered a metabolically independent process used for the uptake of toxic heavy metals. It mainly depends on the electrostatic interactions and different functional groups present on the microbial cell surface (Huang et al., 2019; Dietrich et al., 2021; Han et al., 2023). During the biosorption process, heavy metal ions can passively combine with peptidoglycan on the surface of microbial cells. The biosorption process is highly affected by temperature, pH, ionic strength, size, and amount of absorbent (Timková et al., 2018; Manoj et al., 2020). In contrast, bioaccumulation is a metabolic-dependent process in which cellular energy is used for heavy metal ion uptake. This process depends on the genetic and biochemical properties, environmental conditions, and internal structure of microbial cells (Timková et al., 2018; Yin et al., 2019; Manoj et al., 2020; Sharma et al., 2021). It is influenced by various conditions, such as pH, temperature, and surface charge (Dai et al., 2019; Arce-Inga et al., 2022; Bayuo et al., 2022).

The metal ions can be eradicated from aqueous solutions using live or dead biosorbents. However, the dead biosorbents are preferable over their live counterparts (Hu et al., 2020). Strong and effective heavy metal biosorbents can be obtained by autoclaving bacterial cells as the number of available binding sites increases for more metal ions (Acosta-Rodríguez et al., 2018; Hu et al., 2020; Badawi et al., 2021). The merits of dead biosorbents over their live counterparts may be higher biosorption activity, minimum sludge generation, no requirement of nutrients, and high efficiency (Jin et al., 2016; Hu et al., 2020). In contrast, live biosorbents are characterized by their ability to transfer heavy metal ions into cells, decrease their toxicity, and effectively eliminate them at low concentrations (Dai et al., 2019; Yin et al., 2019; Hu et al., 2020).

Various biological processes have performed new statistical procedures such as definitive screening design (DSD) and artificial intelligence neural network (ANN) (Bayuo et al., 2022; Saber et al., 2023). These methods are highly desirable in bioprocess optimization for leaching heavy metal ions (Ahmad et al., 2014; Elsayed et al., 2022). DSD could be considered an effective method for estimating the main effects of various independent variables affecting the biological process without being biased by the quadratic response or two-variable interaction. Additionally, DSD could be performed for curvilinear and nonlinear influences on the response (Jones and Nachtsheim, 2011; Lin, 2015; Kecić et al., 2018; Jones and Lanzerath, 2021; Elsayed et al., 2022). As a basic tool of machine learning, ANN represents a potential extension of the Response Surface Methodology (RSM) approach. ANN could profoundly afford an empirical model for analyzing the interaction between dependent and independent variables of the biological process with unique output data compared to the RSM approach (Ghritlahre and Prasad, 2018; Saber et al., 2021; Elsayed et al., 2022; El-Shora et al., 2022; Saber et al., 2023; Sabreena et al., 2023). Due to the prettiness ability of ANN to extract and accurately predict the difference between dependent and independent variables over experimental designs, ANN has the advantage of its ability to estimate and predict all kinds of nonlinear functions and models, with no description requirements for an appropriate fitting function, efficient assembly of a successful prediction model, and requiring fewer data. Elsayed et al. (2022) and Saber et al. (2023) reported successful applications of the ANN approach in various biotechnological processes.

There are comparatively few studies that conquer the lead biosorption optimization process using *Bacillus subtilis* by employing a DSD (Kecić et al., 2018; Leong et al., 2020). To the best of our knowledge, no studies in the literature assess the optimal nutritional process using *B. subtilis* biomass for maximizing Pb2+ biosorption efficiency using an ANN. Therefore, a combination of DSD and ANN paradigms was used to optimize various variables influencing the Pb^2+^ biosorption process using *B. subtilis* live biosorbent. The possible biosorption mechanisms of Pb^2+^ using *B. subtilis* were determined using Fourier-transform infrared spectroscopy (FTIR), scanning electron microscopy (SEM), energy-dispersive X-ray (EDX), and transmission electron microscopy (TEM). The influence of adsorbent mass, pH, contact time, and temperature on the Pb^2+^ biosorption efficiency was evaluated by performing different batch biosorption experiments to determine various isotherms and kinetic parameters.

## 2 Materials and methods

### 2.1 Sampling and preparation of Pb^2+^-stock solution

Soil samples were collected from a lead-contaminated area in Cairo, Egypt (El-Sayed et al., 2021; El-Sharkawy et al., 2022). The collected samples were preserved in sterile containers and transported to the laboratory. No definite authorizations were mandatory for these activities or localities. The Pb^2+^-. A salt stock solution was prepared by dissolving 1 g/L of Pb(NO_3_)_2_ (Aldrich, analytical grade) in deionized water. The solution was then filtered, sterilized, and prepared for different final concentrations.

### 2.2 Isolation of Pb^2+^-resistant bacterial isolates

Bacteria exhibiting Pb2+ tolerance were isolated following the method described by Jin et al. (2016) with slight modifications. Briefly, 1 gram from each soil sample was diluted up to 10^-6, and 100 μL of soil suspension was then spread on nutrient agar plates amended with 100 mg/L of Pb2+. The inoculated plates were incubated for 48 h at 37°C. The developed purified cultures were subcultured periodically, preserved on nutrient agar slants, and stored at 4°C until used.

The biosorption susceptibility of the isolated bacteria was determined based on the modified method (Hu et al., 2020). The bacterial isolates were preliminary screened for their capability to grow using modified Lee’s minimal salt medium (LMS) containing (g/l): K_2_HPO_4_ (3), MgSO_4_.7H_2_O (0.7), yeast powder (0.01), (NH_4_)_2_SO_4_ (4), and pH (7.2) supplemented with rising concentrations of Pb^2+^ (10–500 mg/L) (Acosta-Rodríguez et al., 2018; Chintalapudi et al., 2022). The inoculated plates were incubated for 48 h at 37°C. Control samples containing LMS without adding Pb^2+^ were performed under standard assay conditions. The bacterial resistance to heavy metal ions was assessed using the bacteria’s Maximum Tolerance Level (MTL), which is designated as the highest concentration of individual metals supporting bacterial visible growth (Kang et al., 2023; Karn et al., 2023).

### 2.3 Molecular characterization of the highest Pb^2+^-resistant isolate

The most promising bacterial isolate with the highest Pb^2+^-MTL was identified using 16S rDNA gene sequencing (El-Shora et al., 2021; El-Sharkawy et al., 2022; El-Shora et al., 2022; El-Sharkawy and Abbas, 2023). The genomic DNA of bacteria was isolated using an ABT DNA extraction kit. The obtained DNA was amplified using 16S rDNA universal primers for forward (5′–AGA GTT TGA TCC TGG CTC AG–3′) and reverse (5′–GGT TAC CTT GTT ACG ACT–3′). After sequencing the 16S rDNA, the developed amplicon was submitted to GenBank’s Basic Local Alignment Search (NCBI BLASTn similarity search tool).^1^ Fasta sequences of the target 16S rDNA and the closely related sequences in GenBank were imported to Mega-X V 11.0. to construct a phylogenetic tree by the neighbor-joining method with 1,000 bootstrap replication levels of each branch.

### 2.4 Susceptibility of bacteria to Pb^2+^

For live biosorbent preparation, the selected bacterium was inoculated into a sterile nutrient broth medium and was then incubated for 48 h at 37°C on a shaker at 100 rpm. Free-living cells were harvested at the logarithmic growth phase by centrifugation for 10 min at 8,000 × g and 4°C. Subsequently, the bacterium was washed thrice with HCl (150 mM) and sterile deionized water. The bacterial count was attuned to 108 cfu/mL and used as a living biosorbent using a hemocytometer. The bacterial inoculum of 5% (v/v) was introduced to a 50-ml biosorption medium (Acosta-Rodríguez et al., 2018; Choo et al., 2022).

The susceptibility of the bacterial isolate with the highest MTL was determined using certain modifications (Hu et al., 2020). In brief, the bacterial isolate was incubated with various Pb^2+^ concentrations ranging from 0 mg/L to 500 mg/L. The biosorption medium, containing (g/l) yeast extract (5), glucose (5), MgSO_4_.7H_2_O (0.2), K_2_HPO_4_ (0.2), and a pH of 7.0., was prepared and sterilized for 15 min at 121°C. Glucose was individually sterilized using a 0.22 membrane filter and was then supplemented with the sterilized broth medium. The medium with inoculated bacteria was incubated for 48 h at 37°C and 100 rpm. The density of the bacterial cells was determined by monitoring the absorbance at 600 nm. The absorbance of the blank samples containing the same medium with the corresponding Pb^2+^ concentration was immediately monitored after inoculation.

### 2.5 Biosorption process optimization using the DSD

The bioprocess optimization of Pb^2+^ removal using the highest Pb^2 + −^resistant isolate was performed using the **DSD**. A set of 17 runs was performed to detect the significance of the selected physicochemical variables, namely pH, temperature, glucose, yeast extract, MgSO_4_.7H_2_O, and K_2_HPO_4,_ on the Pb^2+^ removal bioprocess. The six continuous variables of the biosorption process were examined at three levels, namely high (+), low (−), and center (0). The actual and coded levels of the DSD matrix are shown in Table 1. The biosorption ability of the potent isolate was used as the assessed response. The full experimental design of 17 trials and the consequent response data are presented in Table 2.

**Table 1:** Definitive screening design, showing operating variables and coded levels affecting the Pb^2+^ removal by *B. subtilis*.

Experimental variable	Variable code	Codified Level		
		−1	0	+1
pH	X_1_	6	7	8
Temperature (°C)	X_2_	20	30	40
Glucose (%)	X_3_	0.5	1	1.5
Yeast extract (%)	X_4_	1.5	2	2.5
MgSO_4_.7H_2_O (%)	X_5_	0.1	0.15	0.2
K_2_HPO_4_ (%)	X_6_	0.1	0.15	0.2

**Table 2:** Matrix of definitive screening design (DSD) for six independent variables affecting Pb^2+^ removal with the consistent experimental and predicted values of DSD and ANN models as the assessed response.

Trials	Variablesa						Pb^2+^ removal response (%)				
	X_1_	X_2_	X_3_	X_4_	X_5_	X_6_	Experimental	DSD		ANN	
								Predicted	Error	Predicted	Error
1	−1	−1	1	1	0	−1	96.44	94.62	1.82	96.42	0.02
2	−1	−1	1	−1	1	1	90.06	90.39	−0.34	90.19	−0.13
3	1	1	1	−1	1	−1	85.88	85.93	−0.05	85.87	0.01
4	1	−1	0	1	1	−1	91.43	91.55	−0.12	91.43	0.00
5	−1	1	0	−1	−1	1	87.44	86.74	0.69	87.49	−0.05
6	−1	−1	−1	1	−1	1	85.54	86.19	−0.65	85.54	0.00
7	−1	1	−1	1	1	0	95.86	94.78	1.07	95.89	−0.03
8	1	1	−1	1	−1	−1	95.99	96.10	−0.01	96.02	−0.03
9	1	−1	−1	0	1	1	91.41	91.63	−0.22	91.12	0.29
10	1	1	−1	−1	0	1	94.05	93.04	0.99	94.90	−0.85
11	1	0	1	1	−1	1	75.91	75.71	0.19	75.91	0.00
12	0	1	1	1	1	1	93.91	95.19	−1.28	93.912	−0.01
13	−1	1	1	0	−1	−1	90.07	91.12	−1.05	90.11	−0.04
14	0	0	0	0	0	0	92.92	93.48	−0.56	92.91	−0.01
15	1	−1	1	−1	−1	0	78.06	77.34	0.70	78.25	−0.19
16	−1	0	−1	−1	1	−1	80.08	80.25	−0.17	80.08	0.00
17	0	−1	−1	−1	−1	−1	84.36	85.28	−0.92	84.36	0.00

^a^The training was assessed by the 11 trials, followed by the next 6 runs for model validation processes. The influential factors level was estimated at high level (+1), low level (−1), and middle level (0). X_1_, pH, X_2_, temperature (°C), X_3_, glucose; X_4_, yeast extract; X_5_, MgSO_4_.7H_2_O (%); X_6_, K_2_HPO_4_ (%).

The developed data were fitted to a polynomial quadratic second-order model, which is illustrated by Equation 1:


(1) $$\zeta =\beta_{0}+\overset{n}{\underset{i=1}{\sum }}\beta_{i}\;X_{i}+\overset{n}{\underset{i=1}{\sum }}X\beta_{ii}\;X_{i}^{2}+\overset{n}{\underset{i=1}{\sum }}\overset{n}{\underset{j=1}{\sum }}\beta ij\;X_{i}X_{j}$$

where ζ is the Pb^2+^ removal (%), $X_{i}$ and $X_{ij}$ are the input operating independent variables, $\beta_{0}$ , $\beta_{ii}\text{,}$ and $\beta_{ij}$ correspondingly represent a regression coefficient of linear, quadratic, and interaction coefficients.

A statistical analysis of the model was carried out using a graphical illustration of the resulting data and an analysis of variance (ANOVA) test to evaluate the significance of each independent operating factor and the adequacy of the model (Singh and Singh, 2016; El-Shora et al., 2022; El-Sharkawy and Abbas, 2023).

To evaluate the efficacy of the model equation, a bacterial inoculum of 5% (v/v, 10^8^ cfu/mL) was injected into a 50-ml biosorption medium amended with 200 mg/L of Pb^2+^. Batch biosorption experiments were performed based on the matrix of DSD with a combination of various operating variables (Jones and Nachtsheim, 2011; Kecić et al., 2018). The inoculated flasks were placed in a thermostatic shaker (100 rpm) at 37°C. By the end of the biosorption process, the cells were collected using centrifugation for 10 min at 8000 × g. Subsequently, the residual Pb^2+^ concentration in the supernatant was determined by an atomic absorption spectrophotometer (Perkin Elmer, United States) based on the method described by El-Beltagi et al. (2024). The Pb^2+^ removal (%) and biosorption rates were, respectively, determined by Equations 2, 3:


(2) $$\mathrm{Lead}\;\mathrm{removal}\;\mathrm{rate}\;\left(\%\right)=\frac{C_{0}-C_{f}\;}{C_{0}}\times 100$$

(3) $$q_{e}=\frac{C_{0}-C_{f}\;}{M}\times V$$

where $C_{0}$ is the initial Pb^2+^ concentration (mg/l), $C_{f}$ is the final Pb^2+^ concentration (mg/l), q_e_ (mg/g) is the equilibrium concertation of Pb^2+^, V is the adsorbate volume (L), and M is the biosorbent weight (g).

### 2.6 Artificial neural network design for modeling Pb^2+^ removal

The developed DSD data from the Pb^2+^-biosorption process was used to feed the predictive artificial neural network (ANN) of the machine learning model (MLM). To optimize the operating factors of the biosorption process, the fully connected ANN was created with a hidden layer in which all nodes had the same hyperbolic sigmoid activation function (NTanH). The ANN-based forecast can judge multiple variables with a minor collection of experiments. Additionally, the ANN prediction can evaluate linear and non-linear interactions without the need for a normal distribution (Leong et al., 2020). The ANN platform was constructed using a 6-h − 1 topology in which the forecast model contains 3 layers. The input layer contains six neurons, namely, pH, temperature, glucose, yeast extract, MgSO_4_.7H_2_O, and K_2_HPO_4_, with the Pb^2+^ biosorption used as the output layer. A hidden layer was performed among the output and input layers with 10 neurons. After the data randomization, the data was employed as two data sets. The first set was for training and consisted of 11 trials, which were employed to reduce the error of forecasting and determine the weight of neurons. The second set was occupied for validation, with six runs to choose the best model and stop training ANN. The holdback procedure was performed at 0.3333 to endorse the developing ANN topology structure. The best fitting of the ANN architecture model using various combinations of the particular ANN parameters was discovered by the performance of the NTanH(10) activation function in the network neurons and hidden layers with various learning rates. The MLM continued until the smallest values of the sum of error’s squares (SES), average absolute deviation (AAD), root mean squared error (RMSE), and the highest variation coefficient (R^2^) among the predicted values and the actual values in the performed paradigm were obtained. The models of DSD and ANN were qualified by comparing the predicted values of both models with the consistent actual experimental results (Narayana et al., 2021; Saber et al., 2023).

### 2.7 Characterization

The homogeneity and surface structure of the selected strain cultured in LMS medium with and without 200 mg/L Pb^2+^ were characterized and photographed under a JEOL JEM-1010 SEM (scanning electron microscope, Tokyo, Japan), attached to an energy-dispersive X-ray detector at 10 keV for elemental analysis. Briefly, the strain was cultured under the optimum conditions of ANN with 200 mg/L Pb^2+^ and then centrifuged twice for 20 min at 5000 × g to harvest the cells. The sample was fixed with glutaraldehyde (2.5%, 40-fold), stored at 4°C for 24 h, washed with phosphate buffer, and dehydrated in increasing concentrations of ethanol (50–00% v/v). The sample was freeze-dried, sputter-coated using gold (20 nm), and subsequently examined using SEM at a 15 kV accelerating voltage. A control sample was prepared by incubating bacterial cells under the same conditions without Pb^2+^ and investigated using SEM.

Samples of the selected strain were prepared in the presence and absence of Pb^2+^ ions using the same conditions for SEM analysis. The samples were scanned in the range of 4,000–500 cm^−1^ using FTIR spectroscopy, and the obtained spectra were analyzed to detect the active groups.

TEM images of control biosorbent and Pb^2+^-loaded biosorbent were inspected using a JEOL JEM − 1,010 transmission electron microscope (Tokyo, Japan). Pb^2+^-untreated and treated cells were suspended in 25% glutaraldehyde for 24 h at 4°C, rinsed with phosphate buffer (pH 7.0), dehydrated using graded series of ethanol, and multiple centrifuged. Thin-sectioned samples were mounted on ultrathin carbon-coated copper grids, stained, and examined under TEM.

### 2.8 Batch experiments

The influences of adsorbent mass (0.1–1.2 g/L), pH (4.0–9.0), contact time (10 min–180 min), and temperature (20–40°C) on the Pb^2+^ removal and biosorption efficiency using the selected biosorbent were tested to determine the optimal conditions (El-Sharkawy and Abbas, 2023). The biosorption experiments were performed in 250-ml Erlenmeyer flasks containing 200 mL of Pb^2+^ solution using the selected biosorbent. All sets of experiments were performed with a standard Pb^2+^ solution of 200 mg/L as the initial concentration unless otherwise mentioned. The biosorption test was conducted at 28°C in an incubator shaker agitated at 100 r/m. After the incubation period, the solution containing bacterial pellets was centrifuged for 10 min at 8,000 × g. The residual concentration of Pb^2+^ ions was determined using an atomic absorption spectrophotometer (Perkin-Elmer, Manchester, United Kingdom), and the Pb^2+^ removal (%) and biosorption rates were calculated using the previous Equations 2, 3. A blank experiment was conducted to detect the possibility of lead biosorption on the walls of the flasks used.

### 2.9 Modeling of equilibrium isotherms and kinetics

The experimental data were fitted using different types of adsorption isotherms. However, the most employed were Langmuir’s and Freundlich’s adsorption isotherm models (Çelebi et al., 2020; Ayub et al., 2021; Elsayed et al., 2022; El-Sharkawy and Abbas, 2023). In this investigation, Langmuir’s and Freundlich’s adsorption isotherms were applied to the experimental data to interpret the equilibrium properties of the adsorption process. The Langmuir’s isotherm was represented by Equation 4, which is used to determine the adsorption parameters.


(4) $$\frac{1}{q_{e}}=\frac{1}{\;K_{L}\;q_{\text{max}}}\;\frac{1}{C_{f}}+\frac{1}{q_{\text{max}}}\text{,}$$

where $q_{e}$ denotes the equilibrium sorption capacity; $q_{\text{max}}$ denotes the maximum biosorption capacity (mg/g); and $K_{L}$ (L/mg) denotes the sorption equilibrium isotherm constant.

The linear form of Freundlich’s isotherm was represented by Equation 5:


(5) $$\log\;q_{e}=\log\;K_{F}+\frac{1}{n}\;\log\;C_{f}\text{,}$$

where the K*_F_* (mg/g) represents the sorption equilibrium constant capacity and n represents the sorption intensity. The $\frac{1}{n}$ value displays the sorption process as either unfavorable ( $\frac{1}{n}$ > 2) or favorable (0.1 < $\frac{1}{n}$ > 0.5).

The biosorption kinetics of the highest Pb^2+^ biosorbent strain were analyzed by the pseudo-first and second-order kinetic models, as pronounced by Equations 6, 7.


(6) $$\ln\left(q_{e}-q_{t}\right)=\ln q_{e}-K_{1}t\text{,}$$

(7) $$\frac{t}{q_{e}}=\frac{1}{K_{2}\;q_{e}^{2}}+\frac{1}{q_{e}}\text{,}$$

where $q_{t}$ is the biosorption rate at time; $K_{1}$ (min^−1^) and $K_{2}$ (g mg^−1^ min^−1^) are the equilibrium rate constants of pseudo-first-order and pseudo-second-order biosorption processes.

The best suitable isotherm and kinetic model for the investigated biosorption process was determined based on the regression coefficient (R^2^).

### 2.10 Experimental design, software, and statistical analysis

The software package JMP Pro V. 17.2 (JMP, SAS Institute Inc., Cary, NC, United States) was used to construct the matrix of DSD and conduct the statistical analysis. The software was also used to perform machine learning models, construct ANN topologies, and plot 3D surface graphs. To enhance the accuracy of the model, machine learning was employed to check the train and validate the experimental data by performing 11 and 6 random runs of the ANN model, respectively. The analysis of biosorption isotherm and kinetics was performed through the Origin V. 2022 software (OriginLab Corporation, Northampton, United States). The experimental data was presented as a means of triplicate runs with SD (standard deviation). Statistical Package Social Science (SPSS) V. 25 was used to analyze the obtained data via ANOVA (analysis of variance) with Tukey’s HDS test for estimating the level of significance at a probability (*P*) of <0.05.

## 3 Results and discussion

### 3.1 Isolation and screening of lead-tolerant bacteria

**Figure 1:**
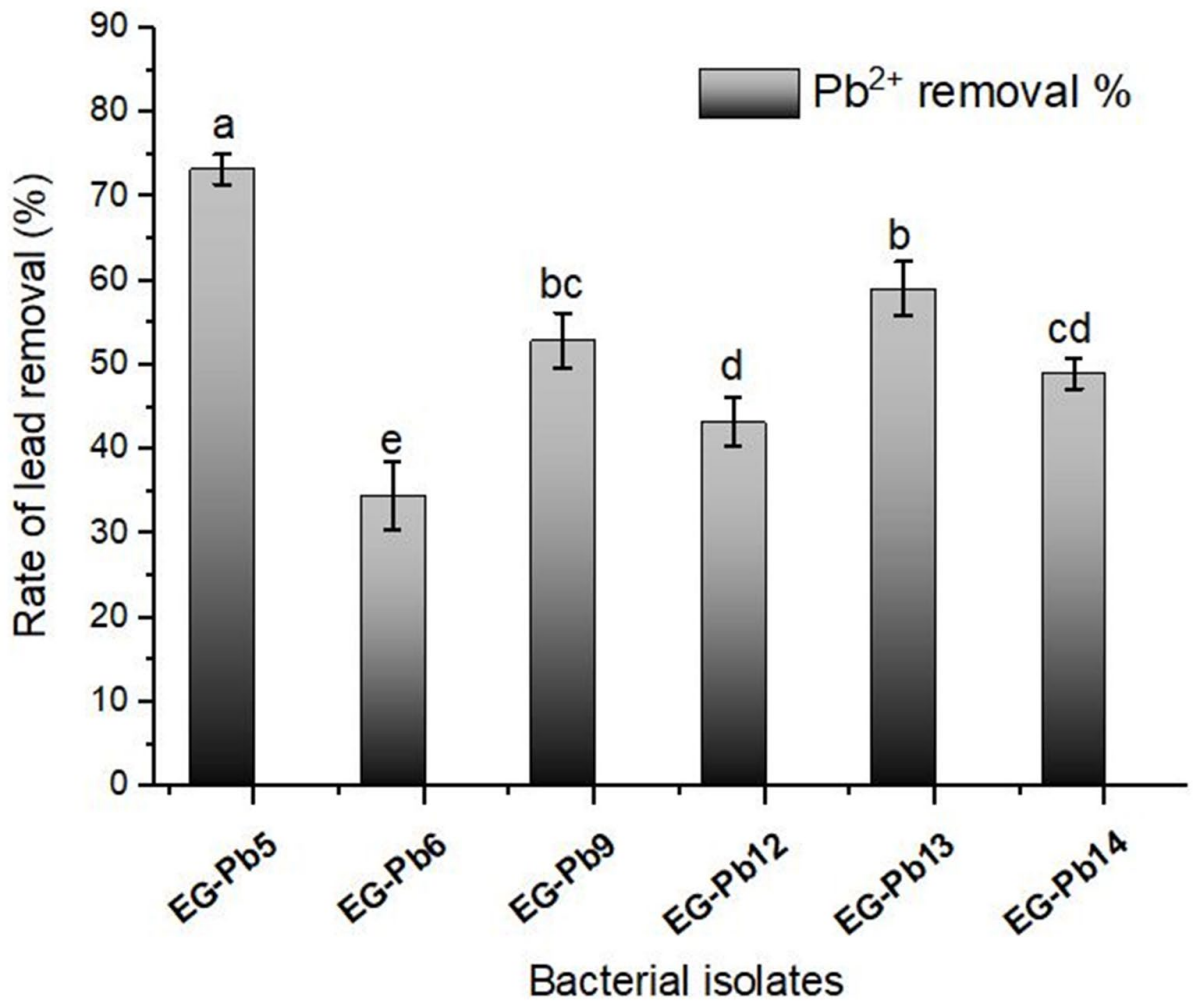
Removal efficiency of Pb^+2^ by the promising bacterial isolates using batch experiments. The experiments were performed at an initial Pb^+2^ concentration of 100 mg/L. All data are represented using three replicates ± standard deviation (SD). Various letters represent a significant difference at a *p*-value of <0.05, according to Tukey’s HSD *post-hoc* test.

The isolation of bacteria that can tolerate Pb^2+^ from the collected soil samples was carried out on a nutrient agar medium containing 100 mg/L of Pb^2+^. A total of fifteen bacterial isolates, namely EG-Pb1 to EG-Pb15, displayed the ability to tolerate the existence of Pb^2+^ in the seeded medium. These bacterial isolates were preliminary screened under varying concentrations of Pb^2+^ and the same environmental conditions. The results showed that only six isolates showed superior resistance against high concentrations of Pb^2+^ based on the maximum tolerance level (MTL). These isolates were then subjected to secondary screening (batch experiments) to select the best biosorbent bacterial isolate using a similar Pb^2+^ ion concentration. Figure 1 clearly shows that EG-Pb5 displayed a higher Pb^2+^-removal percent of 73.3%. Therefore, the isolate EG-Pb5 was selected as the most promising isolate for Pb^2+^ removal from an aqueous solution and subsequently used in further work to optimize various experimental variables to obtain the maximum biosorption of lead using living biomass.

### 3.2 Molecular identification of the best lead-tolerant bacterial isolate and phylogenetic analysis

**Figure 2:**
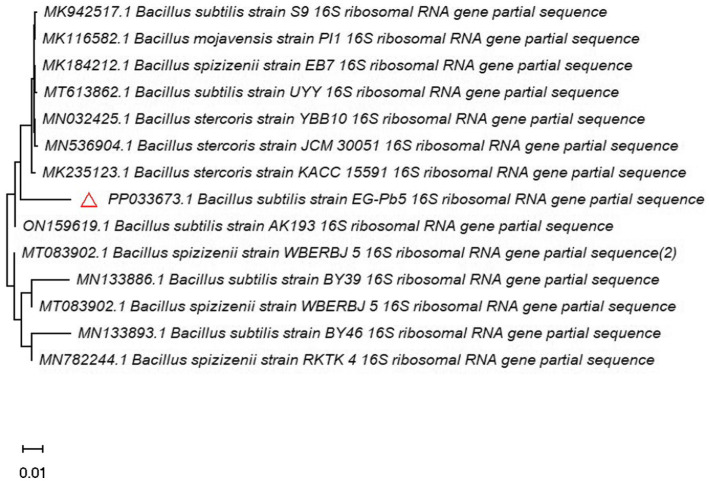
Phylogenetic analysis of the 16S rDNA gene of the *Bacillus subtilis* EG-Pb5 sequence with the retrieved NCBI sequences by Neighbor-Joining tree through MEGA-X 11 software. The symbol (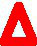) represents the 16S rDNA sequence of the bacterial strain in the present research.

The target strain EG-Pb5 was identified as *Bacillus subtilis* based on the sequence of the 16S rDNA gene, and the NCBI accession number was PP033673.1. Based on the ITS-taxonomic assignment, the evolutionary relatedness of the target strain with the interrelated GenBank species sequences was illustrated in Figure 2. The analysis of the constructed phylogenetic tree showed that the target strain has a 96.64% similar sequence to MK235123.1, ON159619.1, MK942517.1, and MN536904.1.

### 3.3 Susceptibility of *Bacillus subtilis* to Pb^2+^

**Figure 3:**
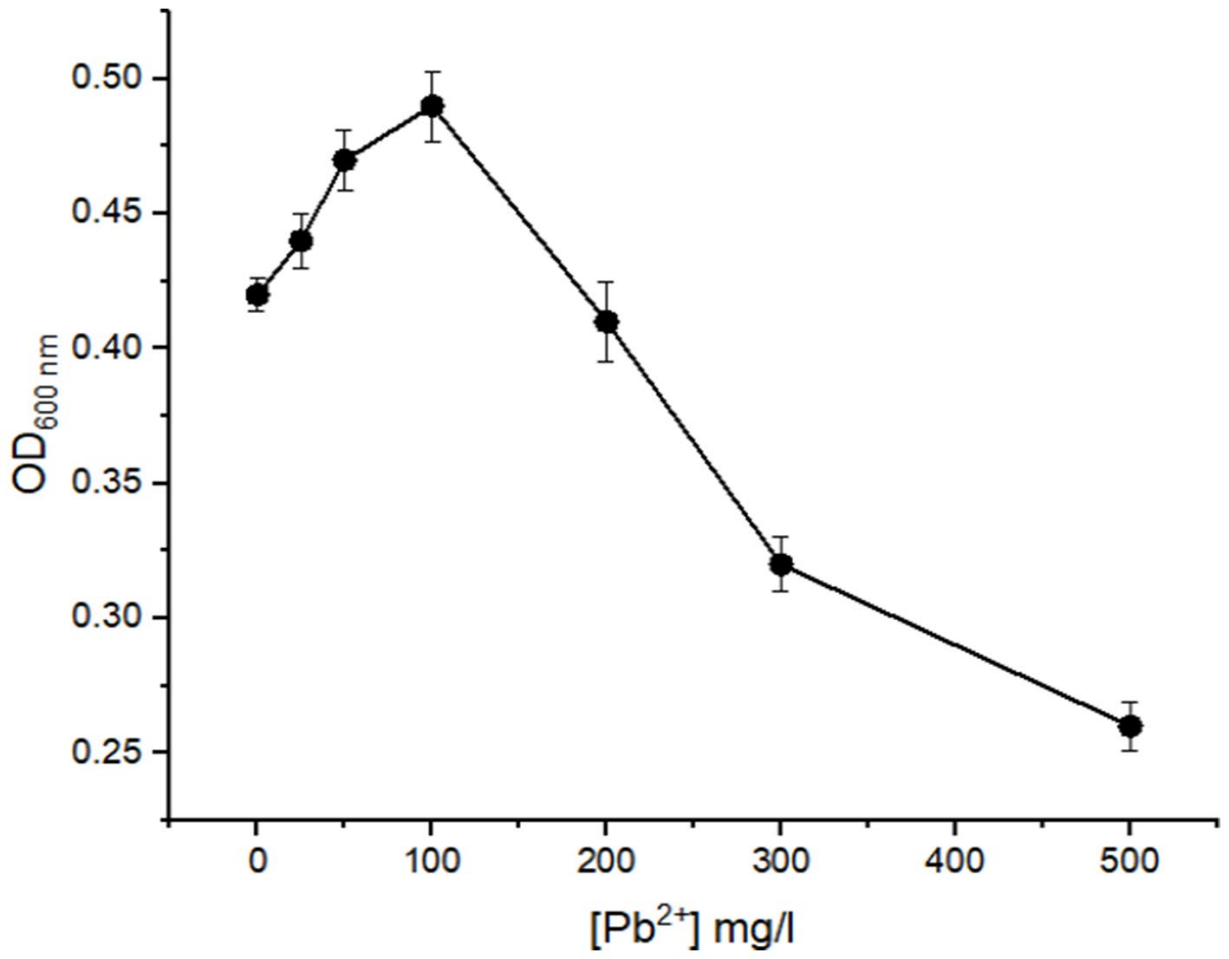
Effect of different initial concentrations of Pb^2+^ on the growth of *B. subtilis*.

The bacterial susceptibility of *B. subtilis* biomass was investigated using different concentrations of Pb^2+^ at 0–500 mg/L. The bacterial growth was then monitored, and the obtained data is presented in Figure 3. The results reveal bacterial growth at all tested Pb^2+^ concentrations. However, *B. subtilis* cells can highly tolerate 200 mg/L of Pb^2+^. The growth of *B. subtilis* is essentially promoted at a relatively low concentration of Pb^2+^ as the transfer of heavy metal ions into the cells occurs and is subsequently mineralized to a non-toxic form (Çolak et al., 2011). The bacterial density was rapidly reduced beyond 200 mg/L, achieving its minimum value at a Pb^2+^ concentration of 500 mg/L. Therefore, 200 mg/L of Pb^2+^ was selected for further study to prevent the inhibition of bacterial growth by heavy metals.

### 3.4 Optimization of the Pb^2+^ biosorption process using the DSD

**Figure 4:**
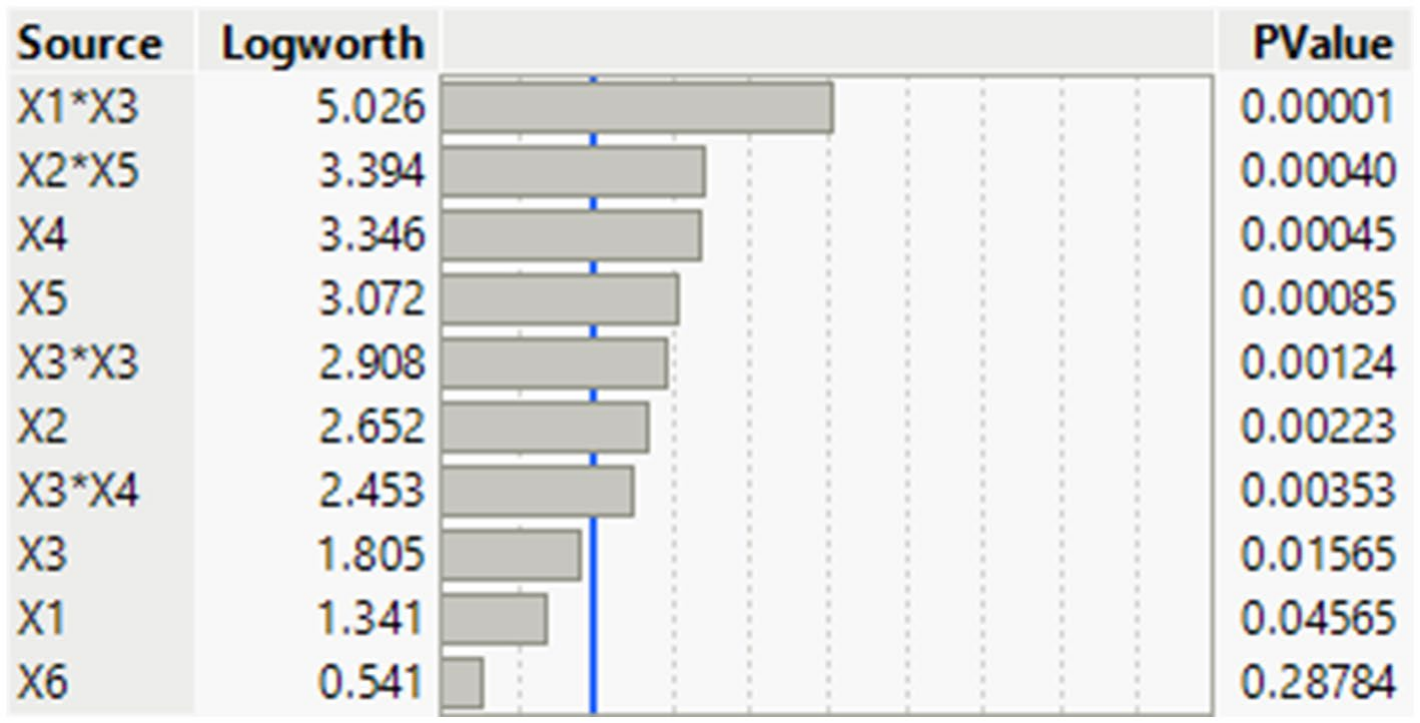
Pareto-chart shows the significance of each operating independent variable on the Pb^2+^ biosorption process using *B. subtilis* biomass. Independent variables beyond the blue line are described as statistically significant factors. X_1_, pH, X_2_, temperature (°C), X_3_, glucose (%); X_4_, yeast extract (%); X_5_, MgSO_4_.7H_2_O (%); X_6_, K_2_HPO_4_ (%).

Six independent operating variables were assessed for their influence on Pb^2+^ biosorption using *B. subtilis*. A set of 17 runs was conducted to enhance the Pb^2+^ biosorption process according to the DSD matrix design, which investigated two physical and four chemical variables. The actual and predicted values were determined as the assessed response during the fluctuation in the combined operating variables on Pb^2+^ biosorption using *B. subtilis* (Table 2). The assessed Pb^2+^ biosorption percentage (response) was statistically evaluated based on probability (*P*) values. A Pareto chart was generated to specify the significance of each independent factor on Pb^2+^ biosorption removal (%). In a descending manner, *p*-values and Longworth are clearly illustrated in Figure 4. The investigated variables exceeded the significance edge level of *p* < 0.05, except for K_2_HPO_4_. Yeast extract was the most significant variable affecting the Pb^2+^ removal percentage. The adaptability and sufficiency of the experimental data model were evaluated by multiple regression analysis using analysis of variance (ANOVA), which provided the Fisher’s *F*-value and model coefficient (R^2^). The correlation between the experimental factors and the values of Pb^2+^ biosorption removal percent (response) was detected from the following regression Equation 8:


(8) $$\begin{array}{l} \zeta =93.48-0.9\;X_{1}+1.85\;X_{2}-1.21\;X_{3}+2.51\;X_{4}+2.23\;X_{5}-\\ 7.92\;X_{1}\times X_{3}+4.34\;X_{2}\times X_{5}-1.87\;X_{3}\;X_{4}-5.70\;{X_{3}}^{2}-0.42\;X_{6} \end{array}$$

where ζ is the Pb^2+^ removal (%), X_1_, pH, X_2_, temperature (°C), X_3_, glucose (%); X_4_, yeast extract (%); X_5_, MgSO_4_.7H_2_O (%); X_6_, K_2_HPO_4_ (%).

The results also showed the significance (*p* < 0.05) of five linear terms (X_1_, X_2_, X_3_, X_4_, X_5_), three interaction terms (X_1_ × X_3_, X_2_ × X_5_, and X_3_ × X_4_), and one quadratic term (X_3_^2^) on the Pb^2+^ biosorption process (Table 3). The overall model is significant, as revealed by the *F*-values of 34.42 and low probability (*p*-value = 0.0002). All the investigated variables displayed a statistically significant effect on the Pb^2+^ biosorption process using *B. subtilis*, with the exception of K_2_HPO_4,_ which had an insignificant negative impact. The lack of fit error showed significant behavior with a model regression coefficient (R^2^) and adjusted R2 of 0.9828 and 0.9543, respectively. Unfortunately, the DSD model is insufficient for predicting Pb^2+^ biosorption using *B. subtilis* biomass, resulting in a significant lack of fit of the model. Therefore, the DSD matrix and the obtained results were further evaluated using the artificial neural network paradigm.

**Table 3:** Analysis of variance for Pb^2+^ removal using *B. subtilis* derived from definitive screening design.

Source		df*	Sum of Squares	*F*-value	Prob > *F*	Remarks
**Overall model**		10	634.7	34.42	0.0002	Sig*
Linear effect	X_1_	1	11.65	6.32	0.0456	Sig
	X_2_	1	47.89	25.97	0.0022	Sig
	X_3_	1	20.54	11.14	0.0157	Sig
	X_4_	1	88.27	47.86	0.0005	Sig
	X_5_	1	69.75	37.82	0.0008	Sig
	X_6_	1	2.51	1.3597	0.2878	
Quadratic effect	X_3_^2^	1	60.36	32.72	0.0012	Sig
Interaction effect	X_2_ × X_5_	1	91.99	49.88	0.0004	Sig
	X_1_ × X_3_	1	345.81	187.52	<0.0001	Sig
	X_3_ × X_4_	1	39.76	21.56	0.0035	Sig
	X_3_ × X_3_	1	60.36	32.72	0.0012	Sig
Lack-of-Fit		3	0.1558	7.89	<0.0001	Sig
Pure error		6	11.06			
R^2^	0.9828					
Adjusted-R^2^	0.9543					

*Sig, significant level; *F*, functions of Fisher’s; R^2^, variation coefficient.

### 3.5 Optimization of Pb^2+^ biosorption process using artificial neural network design

**Figure 5:**
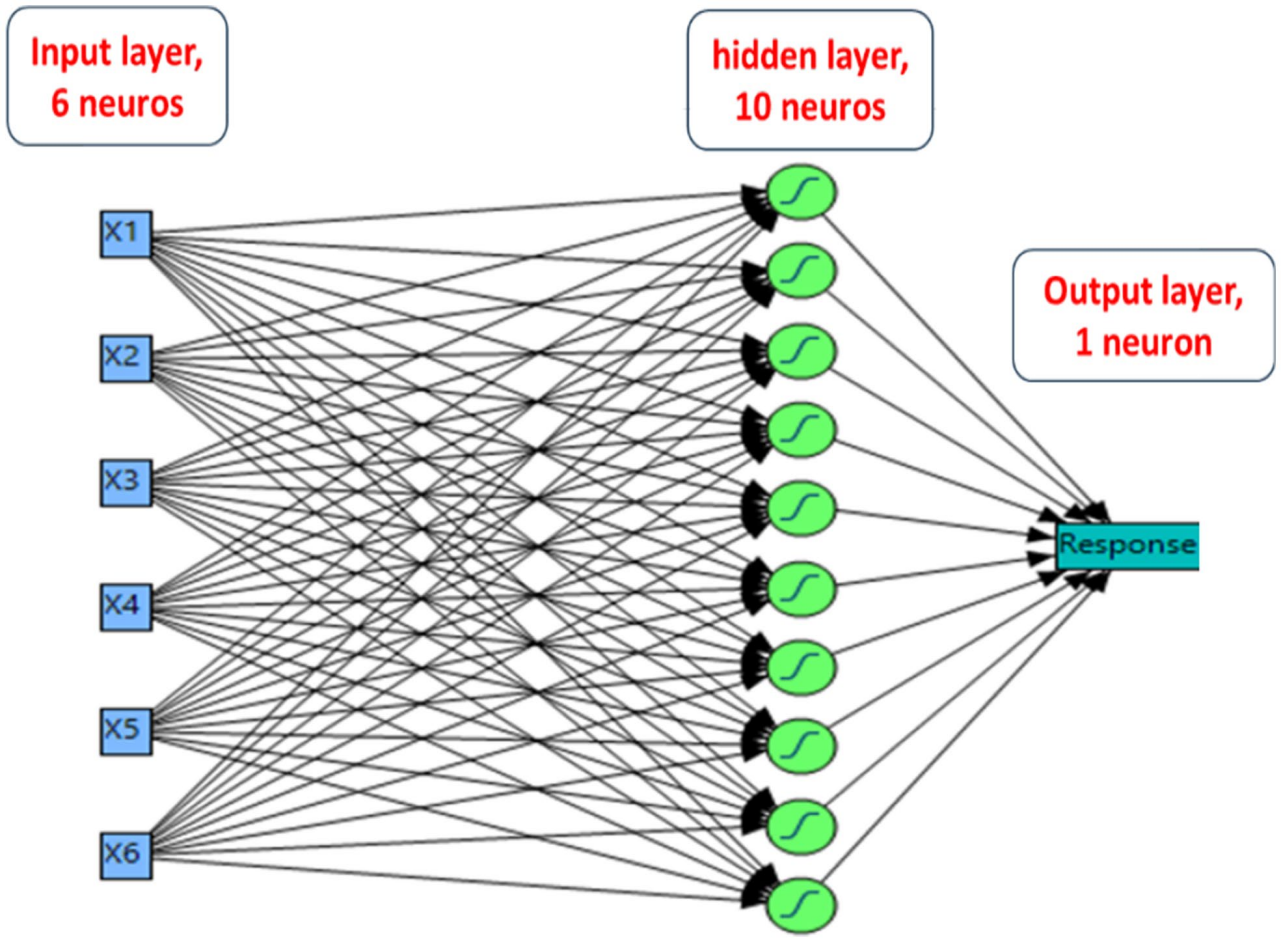
The final proposed artificial neural network of the Pb^2+^ removal by *B. subtilis* biosorbent. The ANN architecture shows a 6-neurons (input layer), 10-neurons (hidden layer), and a single neuron (output layer).

The resulting data from the DSD was employed to develop a forecasting model using the artificial neural network (ANN) paradigm of machine learning models (MLM). A fully connected multilayer feed-forward perceptron of ANN was used for modeling the Pb^2+^ biosorption process. A platform of ANN was performed with six neurons and one hidden layer to determine the superlative architectural structure through the combination of various specific parameters of ANN (Figure 5). The topology of ANN was designated as 6–10-1 with 10 neurons of NTanH(10). The model with the previous conditions was conducted to forecast the response values that could be analogous to the actual output values. The predicted values of the tested ANN were estimated and illustrated in Table 2, whereby the experimental values were considerably closer to the predicted values of ANN when matched with the predicted values of DSD.

### 3.6 Comparison of the DSD and ANN models operation statistics for the efficient Pb^2+^ biosorption process

**Figure 6:**
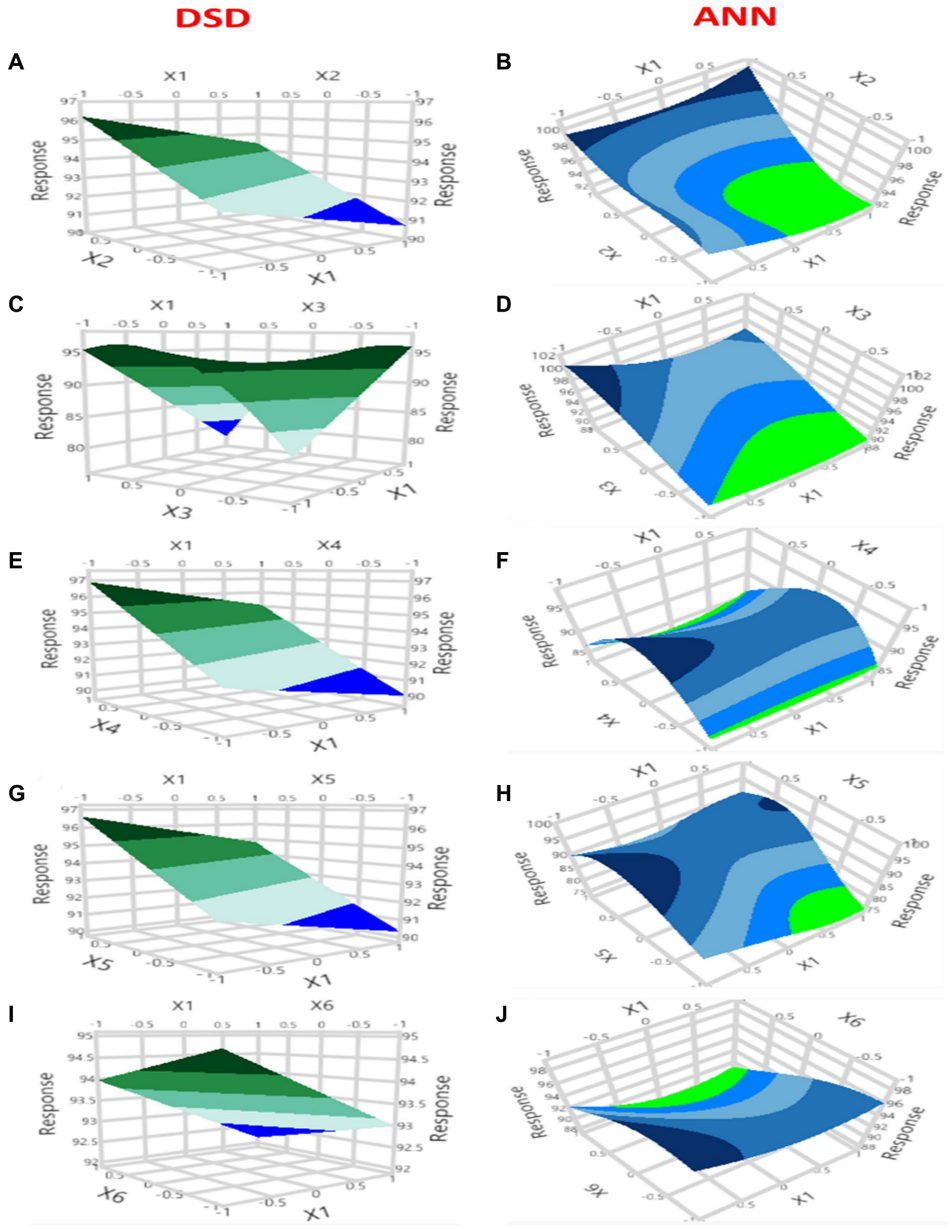
Graphs of three-dimensional surface plots of DSD and ANN for the Pb^2+^ removal by *B. subtilis* illustrate the interactive configuration of each set of the independent variables, keeping the other variables at the middle level.

Three-dimensional response surface graphs were used to determine the effect of pH (an independent variable) as a function on the Pb^2+^ removal by *B. subtilis* when interacting with other different factors: temperature (°C, X_2_), glucose (%, X_3_), yeast extract (%, X_4_), MgSO_4_.7H_2_O (%, X_5_), and K_2_HPO_4_ (%, X_6_). The 3D map plots were created according to the model Equation 1, as illustrated in Figure 6 and Supplementary Figure S1. The Pb^2+^ removal was clearly increased with the decrease of pH to the lower value (around pH = 6.1), while the rise in temperature to 30°C led to a rise in the response point, as shown in Figure 6A. The mutual influence of the investigated X_1_ and X_3_ was illustrated by Figure 6C, where the rise in X_3_ led to a remarkable rise in lead biosorption. The center point of the initial pH and yeast extract shows a positive effect on the Pb^2+^ removal process, where further decreases or increases cause a reduction in the biosorption process (Figure 6E). Figure 6G shows that the increase in the level of MgSO_4_.7H_2_O directed the Pb^2+^ biosorption process to the maximum level. The Pb^2+^ biosorption process was elevated by the rise in the level of K_2_HPO_4_, as illustrated in Figure 6I.

The 3D-surface plots of the ANN paradigm show the initial pH effect upon interaction with five other variables: temperature (°C), glucose (%), yeast extract (%), MgSO_4_.7H_2_O (%), and K_2_HPO_4_ (%) on the Pb^2+^ removal by *B. subtilis*, as shown in Figures 6B,D,F,H,J, respectively. The plots showed various prototypes with an elliptical configuration and not circular, as shown in Figure 6 and Supplementary Figure S2, representing the significant interaction behavior among the operating factors. The Pb^2+^ biosorption by *B. subtilis* increased with the reduction in the pH value. Contrary to the previous result, the biosorption process increased with an elevation in the following variables: temperature, glucose, K_2_HPO_4,_ and MgSO_4_.7H_2_O. The maximum biosorption was determined by the center point of the X_4_ variable (yeast extract).

**Figure 7:**
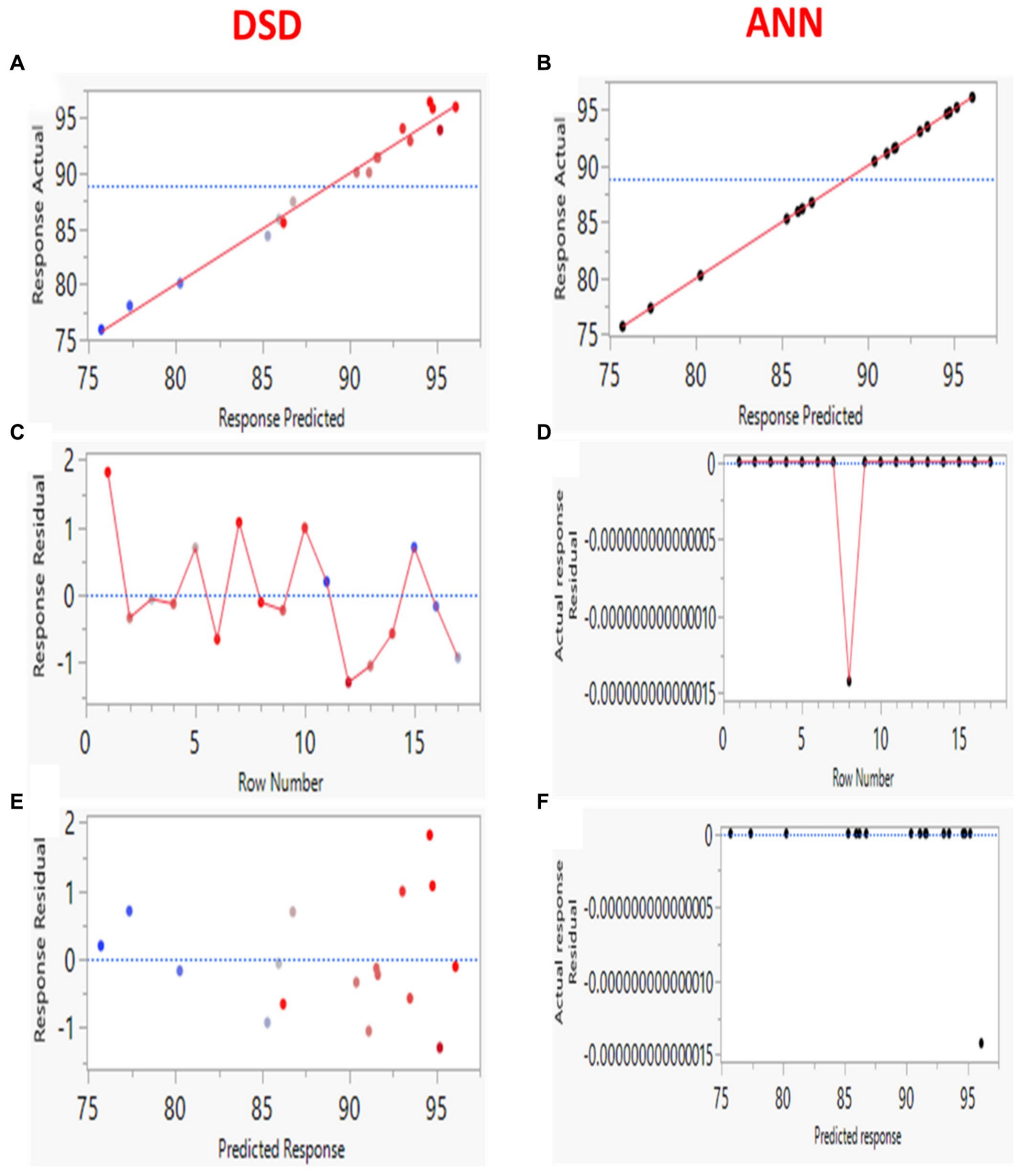
Verification plots of DSD and ANN for the Pb^2+^ removal by *B. subtilis* illustrate the plots of actual vs. predicted values **(A,B)**, internally standardized residuals vs. predicted values **(C,D)**, and residuals vs. predicted values **(E,F)**.

The model’s forecasting ability was further checked by plotting residual values versus predicted ones and standardized residuals against row numbers (Figures 7A–F). The DSD results showed a random distribution of the residuals along both sides of the 0-axis with five extreme outliers (Figures 7A,C,E). The lack of fit of the model is significant; therefore, the DSD model is insufficient for forecasting Pb^2+^ biosorption using *B. subtilis* biomass. In contrast, the ANN model showed an equal distribution of the residual points, which spread very closely to the 0-axis with linearity (Figures 7B,D,F). The accuracy of both models was also evaluated by plotting experimental versus predicted results. The ANN prediction points were found to be near the perfect forecast, suggesting the model prediction can faithfully approach the experimental values. The ANN model displayed lower values for RMSE, SES, and AAD and higher values for R^2^, compared with the same values of the DSD model (Supplementary Table S1). Therefore, the higher probability indicates that the ANN model paradigm can be performed to fit the experimental data with a higher forecasting aptitude than the DSD model.

### 3.7 Validation of DSD and ANN

To ensure the validation and fitness of the software prediction, laboratory experiments were conducted using the optimal conditions, which were determined using the desirability function. The optimal conditions were found to be pH 6.1, temperature 30°C, glucose 1.5%, yeast extract 1.7%, K_2_HPO_4_ 0.2%, and MgSO_4_.7H_2_O 0.2%. The estimated predicted response (Pb^2+^ removal by *B. subtilis*) is remarkably close to the experimental values (Supplementary Table S1), indicating the reliability, meaningfulness, and effectiveness of the optimal conditions compared to DSD, which displayed an unusual value of 115.2%. The results showed the feasibility of the ANN predictive tools in optimizing Pb^2+^ removal by *B. subtilis*. Previous research by Venil et al. (2011) reported that optimizing different variables affecting the Cr^2+^ removal using *Bacillus* REP02 was highly desirable. The process optimization was conducted using the Box–Behnken design, and the developed results showed closely related behavior among the experimental and predicted values. El-Naggar et al. (2020) conducted face-centered central composite design (FCD) to optimize the fermentation conditions of *Pseudomonas alcaliphila* NEWG-2 for the maximum removal of Cr^6+^. The removal percent was found to be 96.6% by growing the bacterium for 48 h at a pH of 7.0 in a medium containing g/l glucose (4.9) and yeast extract (5.6), as determined using FCD.

### 3.8 SEM and EDX analyses

**Figure 8:**
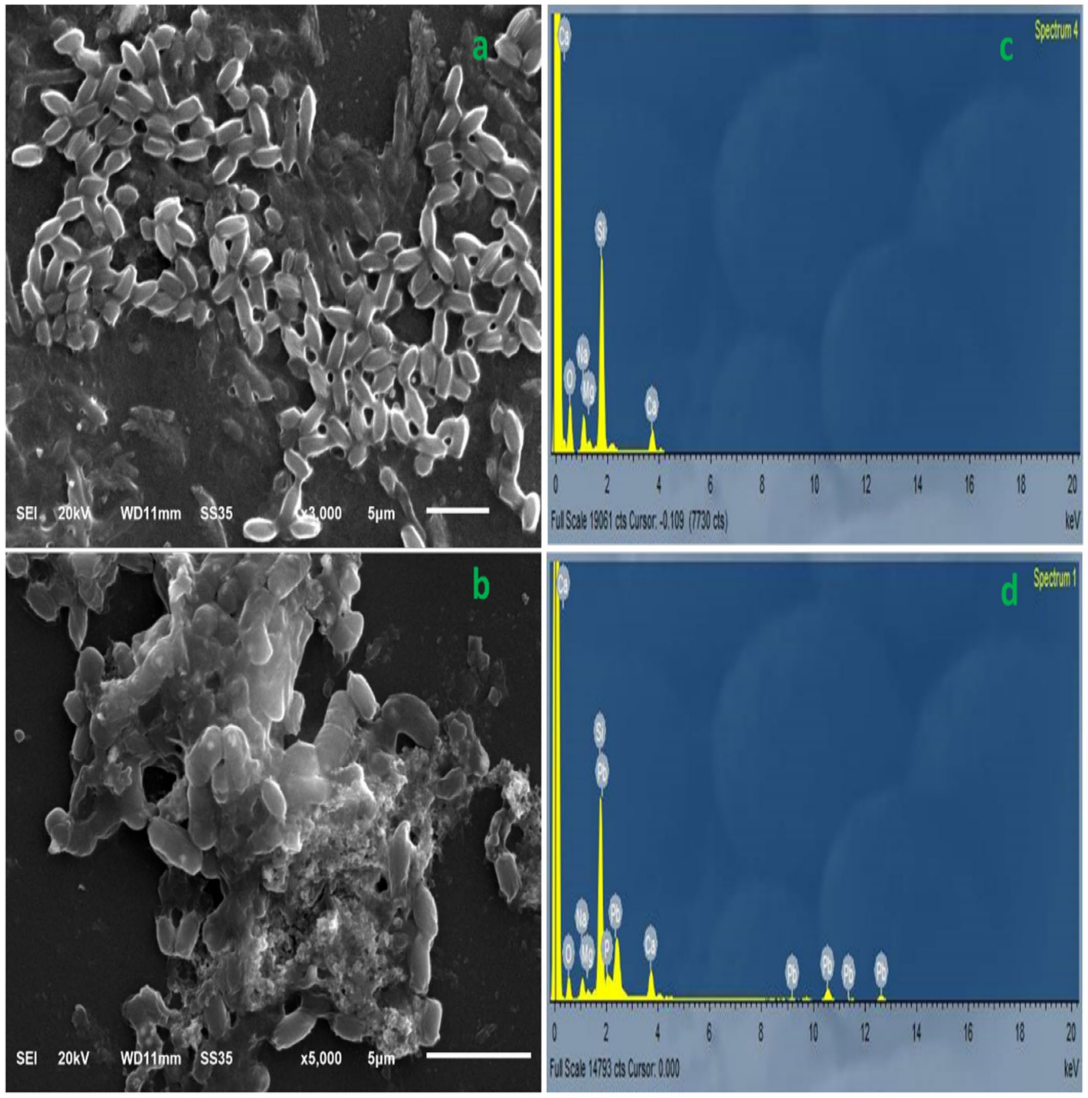
Scanning electron microscopy micrograph illustrating the normal cells of *B. subtilis* **(A)** and the modification after Pb2+ ions biosorption **(B)**. Electron dispersive spectroscopy of the native cell elements of *B. subtilis* live biosorbents **(C)** and Pb2 + −loaded biosorbents **(D)** shows the appearance of Pb^2+^ ions after the process of biosorption.

The morphological variation and surface texture of *B. subtilis* grown under non-metal and Pb^2+^-treated conditions are shown in Figure 8. Before the biosorption process, the micrographs of *B. subtilis* cells illustrate a smooth surface and its growth with a typical bacillus-shaped morphology (Figure 8A). In contrast, the morphology and size of *B. subtilis* biosorbent live cells treated with Pb^2+^ ions displayed remarkable fluctuations. A reduction in cell size, ruptured and rough surfaces, some cell deformation, and Pb^2+^ biosorption were observed, as illustrated in Figure 8B. The EDS spectra of the biosorbent before and after Pb^2+^ sorption confirmed the presence of Pb^2+^ ions in the biosorbent biomass, while Pb^2+^ ions were absent in the EDX spectra of the control biomass (Figures 8C,D). The major elements of control cells were O (46.62%), Na (9.73%), Mg (2.18%), Ca (6.14%), and Si (35.33%). However, those of metal-treated live cells were O (34.75%), Na (6.12%), Mg (1.30%), Ca (5.37%), Si (30.00%), and Pb^2+^ (22.46%). Similar findings were reported by Hu et al. (2020) for *Rhodococcus* sp. HX-2 grown under Pb^2+^-treated conditions.

### 3.9 FTIR analysis

**Figure 9:**
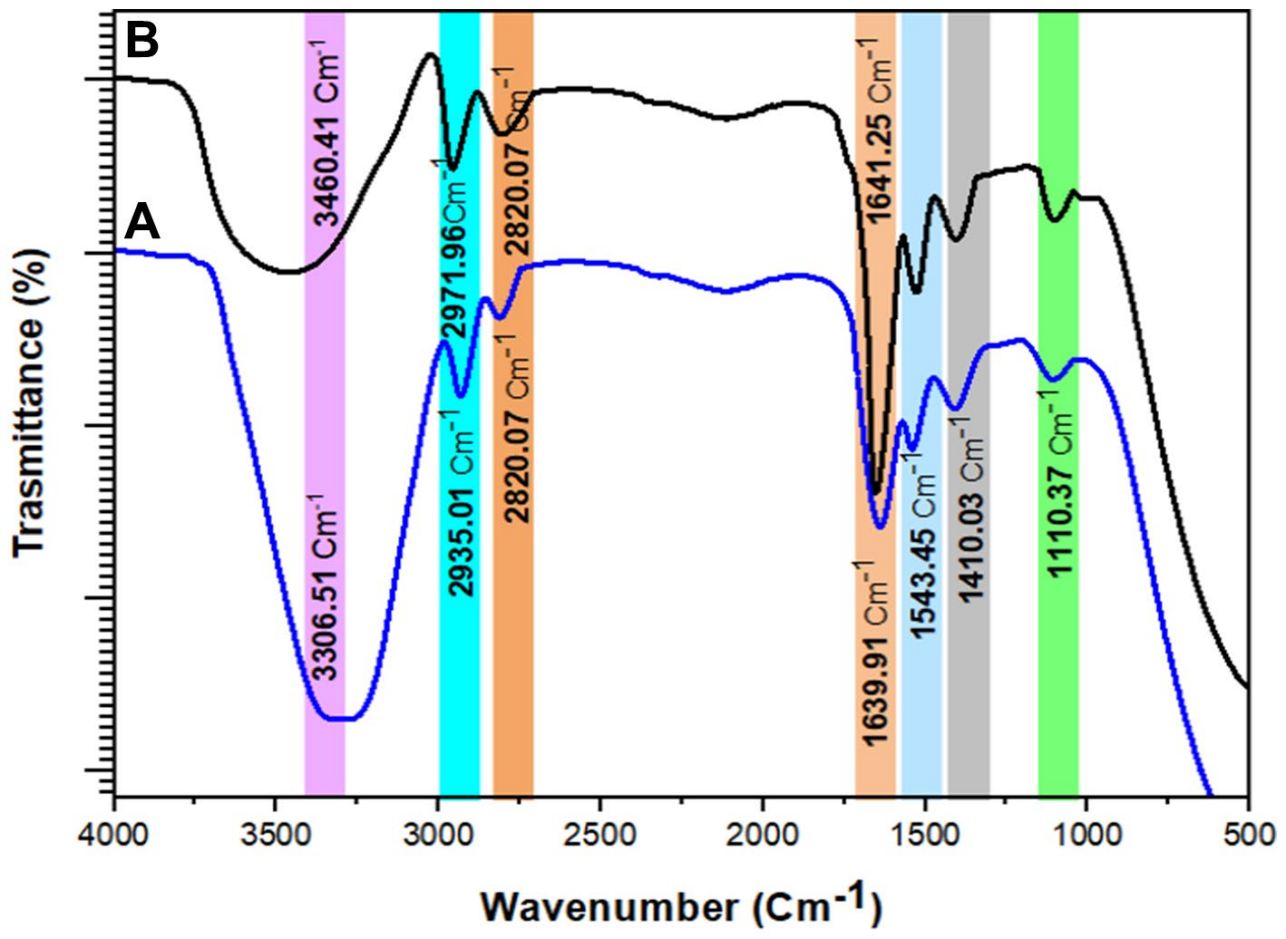
The FTIR of **(A)** native live biosorbents without Pb2+ and **(B)** live biosorbents loaded with Pb^2+^, whereby [Pb^2+^] = 200 mg/L.

The FTIR spectral analysis of *B. subtilis* before and after Pb^2+^-biosorption was performed in the range of 500–4,000 cm^−1^ wavenumber, as shown in Figure 9. The biosorption process was commonly attributed to various changes in the cell surface characteristics, cell morphology, and functional group frequency with the development of other bonds. A characteristic broadband was observed at 3306.51 cm^−1^, hinting at the overlapping of stretching vibrations for the –OH and –NH groups. The absorption bands at 2935.01 cm^−1^ and 2820.07 cm^−1^ could be attributed to the O–H and C–H stretching vibrations of carboxylic, alcohol, and alkane groups. The bands at 1639.91 and 1543.45 could be, respectively, attributed to the amide II bond in the protein bonds and the C–N stretching vibration. The absorption band at 1110.37 cm^−1^ was associated with the stretching vibration of C–O of carboxylic acid and alcohol. The FTIR analysis of Pb^2+^-loaded biomass shows a shift in the band positions, indicating the involvement of the main functional groups (–NH, –OH, C–O) in Pb^2+^ biosorption, as illustrated in Figure 9B.

### 3.10 TEM analysis

**Figure 10:**
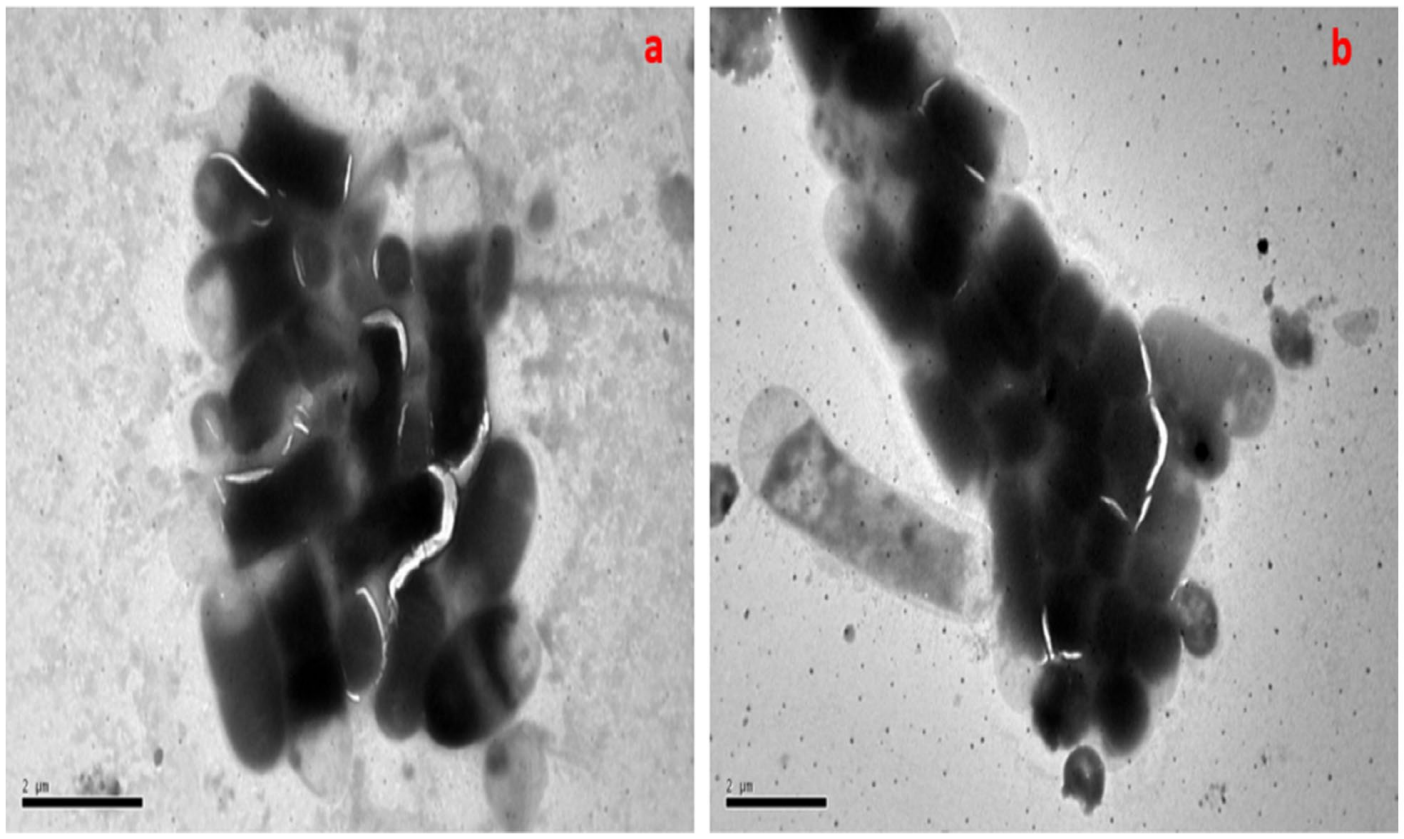
TEM micrographs of *B. subtilis* before **(A)** and after incubation with Pb^2+^ **(B)**.

Transmission electron microscopy (TEM) was conducted on *B. subtilis* live biomass before and after Pb^2+^ biosorption using the optimum conditions. Figure 10A clearly illustrates the control *B. subtilis* biosorbent electron micrograph, which exhibited a typical intact cell with a normal appearance. A micrograph of live biosorbent after Pb^2+^ sorption displayed an intact morphology with numerous dots in the cells owing to Pb^2+^ accumulation, as shown in Figure 10B. Various appearances and statuses of biosorbent cells were determined before and after exposure to pb^2+^ ions, as reported by Deng et al. (2019) and Hu et al. (2020).

### 3.11 Batch biosorption experiments

Batch biosorption experiments were conducted using a solution of 200 mg/L of Pb2+ to investigate the biosorption characteristics of *B. subtilis* live biomass.

#### 3.11.1 Effect of initial biosorbent dosage

**Figure 11:**
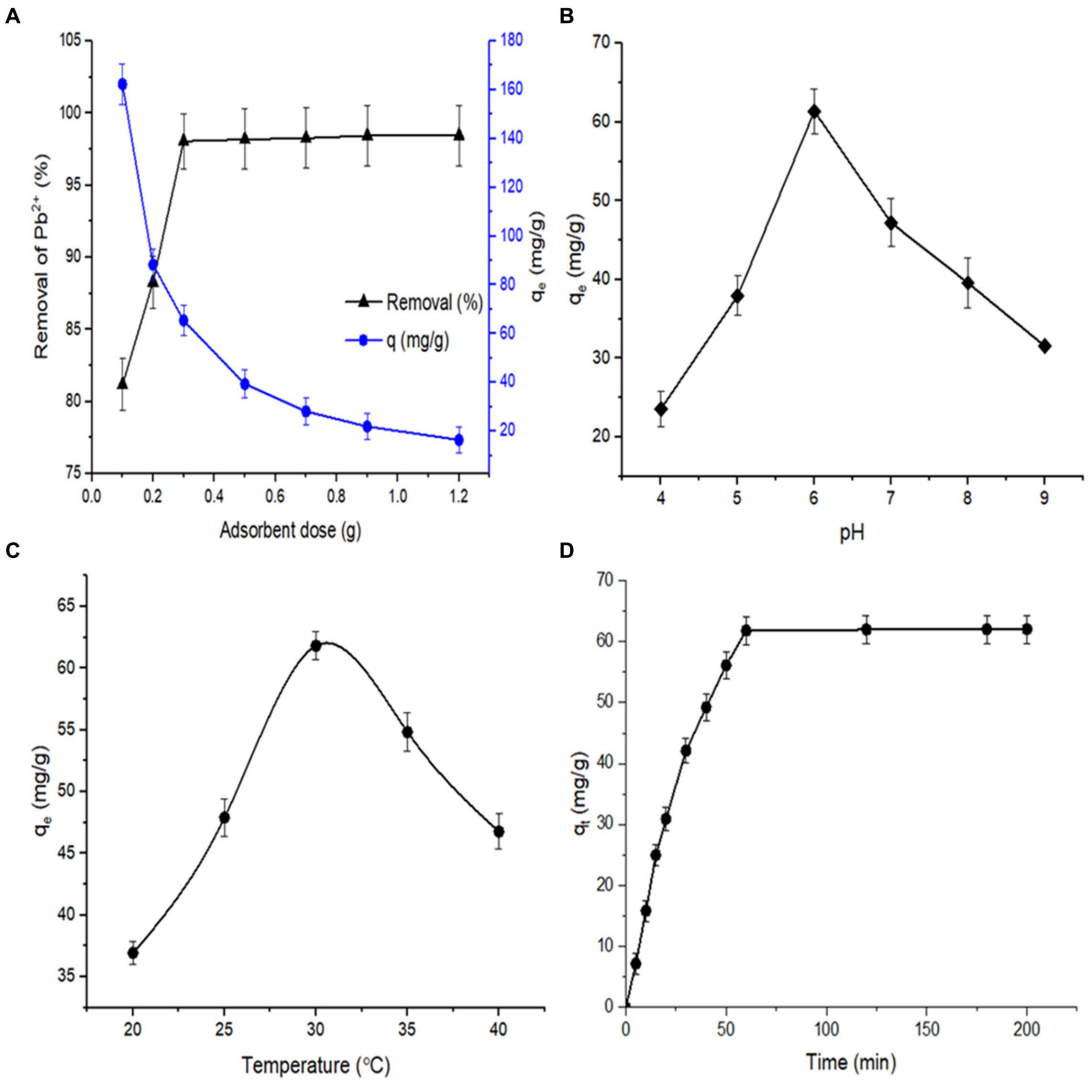
**(A)** Effect of adsorbent dosage on biosorption capacity and removal rate of Pb^2+^ by *B. subtilis* living biomass (at pH 6.0, initial concentration 200 mg/L, contact time 60 min; temperature 30°C, 100 mL volume, 100 rpm). **(B)** Effect of pH on biosorption capacity of Pb^2+^ by living *B. subtilis* biomass (at temperature 30°C, initial concentration 200 mg/L, contact time 60 min; 100 mL volume, 100 rpm). **(C)** Effect of different temperatures on biosorption capacity of Pb^2+^ by living *B. subtilis* biomass (at pH 6.0, initial concentration 200 mg/L, contact time 60 min; 100 mL volume, 100 rpm). **(D)** Effect of contact time on biosorption capacity of Pb^2+^ by living *B. subtilis* biomass (at initial Pb^2+^ concentration 200 mg/L, pH 6.0, biosorbent dosage 0.3 g, contact time 60 min; temperature 30°C, 100 mL volume, 100 rpm).

The optimum adsorbent dose was determined by studying the effect of different biosorbent doses on Pb^2+^ biosorption. Figure 11A shows that the removal (%) gradually increased as the biosorbent dosage rose from 0.1 to 0.3 g/L. However, the biosorption efficiency decreased. Beyond the 0.3 g biosorbent dose, there is no noteworthy influence on Pb^2+^ removal efficiency. The availability of more binding sites rises with increasing adsorbent doses, and therefore, an increase in the Pb^2+^ removal percentage was observed. However, a further rise in the biosorbent dose at a certain metal ion concentration is unnecessary for a sufficient biosorption process. The high-speed superficial biosorption of metal ions onto bacterial cells results in an extreme reduction in the free biosorption sites. Consequently, the biosorption capacity (q_e_) of Pb^2+^ is reduced (Abu-Danso et al., 2020; Hussain et al., 2020; Ayub et al., 2021).

#### 3.11.2 Effect of pH

To evaluate the influence of various pH values on Pb^2+^ removal effectiveness, the biosorption experiments were conducted at six different pH values, i.e., 4, 5, 6, 7, 8, and 9, and the obtained results are illustrated in Figure 11B. The biosorption capacity steadily increased during the elevation in pH values from 3.0 to 6.0, with corresponding q_e_ values of 23.57 mg/g and 61.8 mg/g, recording the highest biosorption capacity at pH 6.0. The Pb^2+^ biosorption power was noticeably reduced from pH 7.0 onward. The lower biosorption capacity at lower pH can be ascribed to the high concentration of H^+^, resulting in the protonation of the active binding site and, subsequently, the occupancy of the competitive biosorption site for Pb^2+^ and H^+^ (Huang et al., 2019; Hu et al., 2020). At higher pH, the biosorption power was reduced due to the reaction of Pb^2+^ and OH^−^ in the solution, producing Pb(OH)_2_. Subsequently, the number of adsorbable Pb^2+^ ions in the working solution was decreased (Ghorbani et al., 2022; Mathivanan et al., 2023). Therefore, it is established that the biosorption process is highly dependent on the pH value of the solution, as it affects not only the functional groups on the microbial cell wall surface but also the chemicals inside the microbial cell and the availability of free metal ions in the solution (Acosta-Rodríguez et al., 2018; Ayub et al., 2021; Sharma and Shukla, 2021).

#### 3.11.3 Effect of temperature

The influence of temperature on the biosorption capacity of Pb^2+^ was tested at five temperatures, and the obtained data is illustrated in Figure 11C. The biosorption capacity increased with a rise in temperature from 20°C to 30°C, reaching its maximum value at 30°C. However, the further elevation in incubation temperature leads to a reduction in the Pb^2+^ uptake by *B. subtilis* biomass. This may be attributed to the exothermic property of metal uptake reactions, ion transudation into working solutions, and the destruction of heavy-metal binding sites on the bacterial surface (Wang et al., 2023).

#### 3.11.4 Effect of contact time

The influence of contact time on the lead biosorption capacity using *B. subtilis* living biomass was evaluated at the initial concentration of lead (200 mg/L), pH of 6.0, and 0.3 g biosorbent dose at ambient temperature, and the developed data are presented in Figure 11D. It is observed that the biosorption capacity is gradually increased during the elevation of contact time at the sorbent/contaminant surfaces. The biosorption rate of *B. subtilis* biomass displayed more than 61.8 mg/g at the first 50 min, reaching the equilibrium rate after 60 min of contact time, after which the value of q_e_ became unchanged. Therefore, the contact time was optimal at 60 min in the consequent experiments. The lead sorption process consisted of two stages: an initial rapid biosorption phase and a second slow biosorption phase for Pb^2+^ metal uptake. The rapid biosorption capacity at the initial stage may be due to the accessibility of all active binding sites for the Pb^2+^ ions; thereafter, the adsorbent surface became almost occupied with the Pb^2+^ ions during further increases in time, resulting in a very quiet rise in the metal biosorption rate. Other researchers have described similar findings (Hu et al., 2020; Ayub et al., 2021; Chintalapudi et al., 2022).

#### 3.11.5 Effect of initial concentration

**Figure 12:**
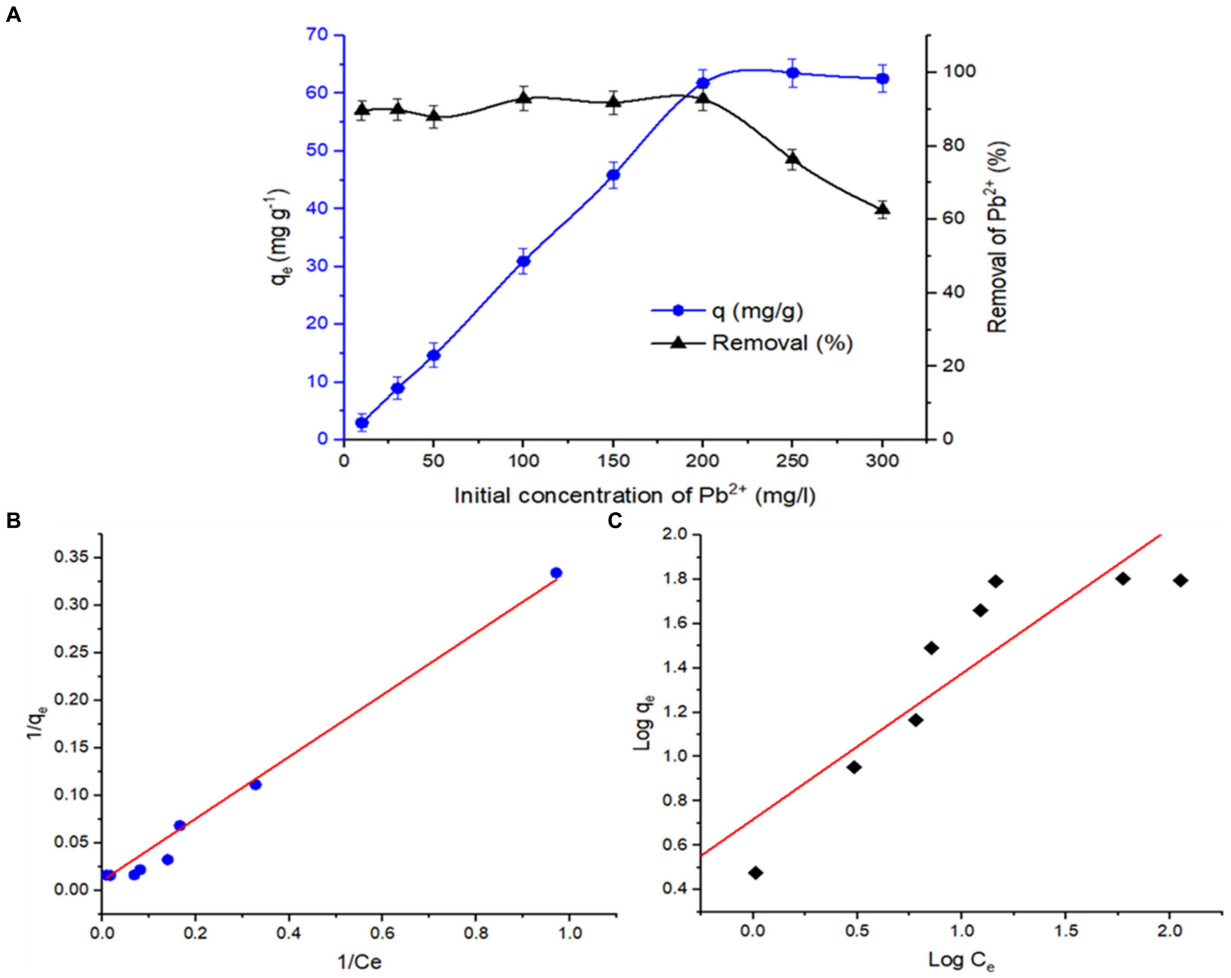
**(A)** Effect of initial Pb^2+^ concentration on biosorption capacity and removal rate of Pb^2+^ by living *B. subtilis* biomass (at pH 6.0, biosorbent dosage 0.3 g, contact time 60 min; temperature 30°C, 100 mL volume, 100 rpm). **(B)** Langmuir and **(C)** Freundlich isotherms for Pb^2+^ biosorption using living *B. subtilis* biomass (at pH 6.0, biosorbent dosage 0.3 g, initial Pb^2+^ concentration, 200 mg/L, contact time 60 min; temperature 30°C, 100 mL volume, 100 rpm).

To evaluate the effect of the initial Pb^2+^ concentration on the biosorption capacity and biosorption rate (%) of *B. subtilis* living biomass, batch experiments were conducted in a shaker at 30°C with a pH of 6.0 and a 0.3 g biosorbent dose with a metal concentration of 10–300 mg/L. It was noticed that the biosorption rate sharply increased from 3.19 mg/g to 61.8 mg/g on increasing the Pb^2+^ concentration in the range of 10–200 mg/L (Figure 12A), which may be due to an increment in driving force related to a high metal concentration in solution and high availability of sorption sites on biosorbent surfaces with less competition (Chintalapudi et al., 2022). Subsequently, a plateau was observed with a further increase in metal concentration in the working solution. This behavior may be attributed to the saturation of the binding sites on the solid–liquid interface, which produces a competition for binding Pb^2+^ ions to the active sites. In contrast, the Pb^2+^ removal rate decreased with the initial metal concentration in the solution being raised, indicating a reduction in the binding affinity of metal ions to biosorption sites by increasing the initial Pb^2+^ concentration. Therefore, the Pb^2+^ concentration of 200 mg/L was selected as optimal for the subsequent experiments to ensure the balance of the biosorption process.

### 3.12 Modeling of biosorption isotherms

The experimental data were applied to various isotherms to obtain insight into the existing equilibrium mechanism among the sorbent surface and the metal ion concentration in the solution (Çelebi et al., 2020; El-Sharkawy and Abbas, 2023). In the present study, Langmuir and Freundlich isotherm models were studied (Figures 12B,C), and the calculated parameters are depicted in Table 4. Based on the values of the regression coefficient (R^2^), the experimental data were better fit with Langmuir’s adsorption isotherm (R^2^ = 0.9910) than Freundlich’s adsorption isotherm (R^2^ = 0.7922) as illustrated in Figure 12B, hinting at the occurrence of a monolayer of biosorption Pb^2+^ ions on the biomass surface of *B. subtilis*. The maximum absorption capacity (q_max_) was 61.8 mg/g for surface *B. subtilis* biomass against Pb^2+^. The q_e_ values of the biomass surface of *B. subtilis* against Pb^2+^ ions have been matched with the results of various sorbents described in the literature, as shown in Table 5. The Langmuir’s isotherm describes the homogeneity of the biosorbent surface with specific active adsorbent sites, which supports the formation of the monolayer with the sorbed metal ions, whereby the adsorbed metal ions bind to a single active site without forming any interaction with neighboring biosorbent metal ions (chemisorption) (Ayub et al., 2021; El-Sharkawy and Abbas, 2023). Freundlich’s adsorption isotherm supports that the biosorbent surface is heterogeneous, whereby an interaction occurs among the biosorbent metal ions and adjacent ones, demonstrating the physisorption (Sierra-Trejo et al., 2020). The separation factor value (R_L_) is less than 1, hinting that the biosorption is favorable. In the experimental results of other biosorbents reported in the literature, Pb^2+^ sorption is modeled and better fitted by the Langmuir (Badawi et al., 2021; Nguyen et al., 2022) or Freundlich (Hu et al., 2020; Sheikh et al., 2021) isotherms.

**Table 4:** Isotherms constants and Kinetic parameters for the removal of Pb^2+^ ions by *B. subtilis* biomass.

(a) Isotherm model	Langmuir isotherm			Freundlich isotherm		
	q_max_ (mg/g)	*K_L_* (L/mg)	R^2^	K_F_ (mg/g)	*n*	R^2^
	97.08	0.0316	0.991	5.22516	1.521	0.792

(b) Kinetic model	Pseudo-first-order			Pseudo-second-order		
	q_e_ (calc.) (mg/g)	K_1_ (min^−1^)	R^2^	q_e_ (calc.) (mg/g)	K_2_ (g/mg/min)	R^2^
	11.10	0.0002	0.701	62.695	0.02793	0.999

**Table 5:** Comparison between the equilibrium time, biosorbent dosage, initial Pb^2+^ concentration, maximum adsorption capacity using living *B. subtilis* biomass with other biosorbents.

Biosorbent biomass	pH	Equilibrium time	Biosorbent dosage (g/l)	Ci^a^	q_max_ (mg/g)	Reference
*B. subtilis*	6.0	60 min	0.3	200	62.69	Current study
*Bacillus cereus*	5.5	24 h	3	100	36.7	Pan et al. (2007)
*B. pumilus*	6.0	80 min	1	75	28.06	Çolak et al. (2011)
*B. cereus*	6.0	80 min	1	75	22.1	Çolak et al. (2011)
*Arthrobacter* sp. *25*	5.75	25 min	9.9	108.79	9.6	Jin et al. (2016)
*Lactobacillus brevis*	6.0	12 h	3	100	53.632	Dai et al. (2019)
*Brevibacillu*	5.5	12 h	1	150	128.58	Chintalapudi et al. (2022)
*Bacillus cereus* BPS-9	8.0	72 h	1	100	193.93	Sharma and Shukla (2021)
*Bacillus cereus*	6	10 min	5	150	60.9	Li et al. (2023)

^a^ $C_{i}$ , initial pb^2+^concentration; $q_{\text{max}}$ denotes the maximum biosorption capacity (mg/g).

### 3.13 Biosorption kinetics

**Figure 13:**
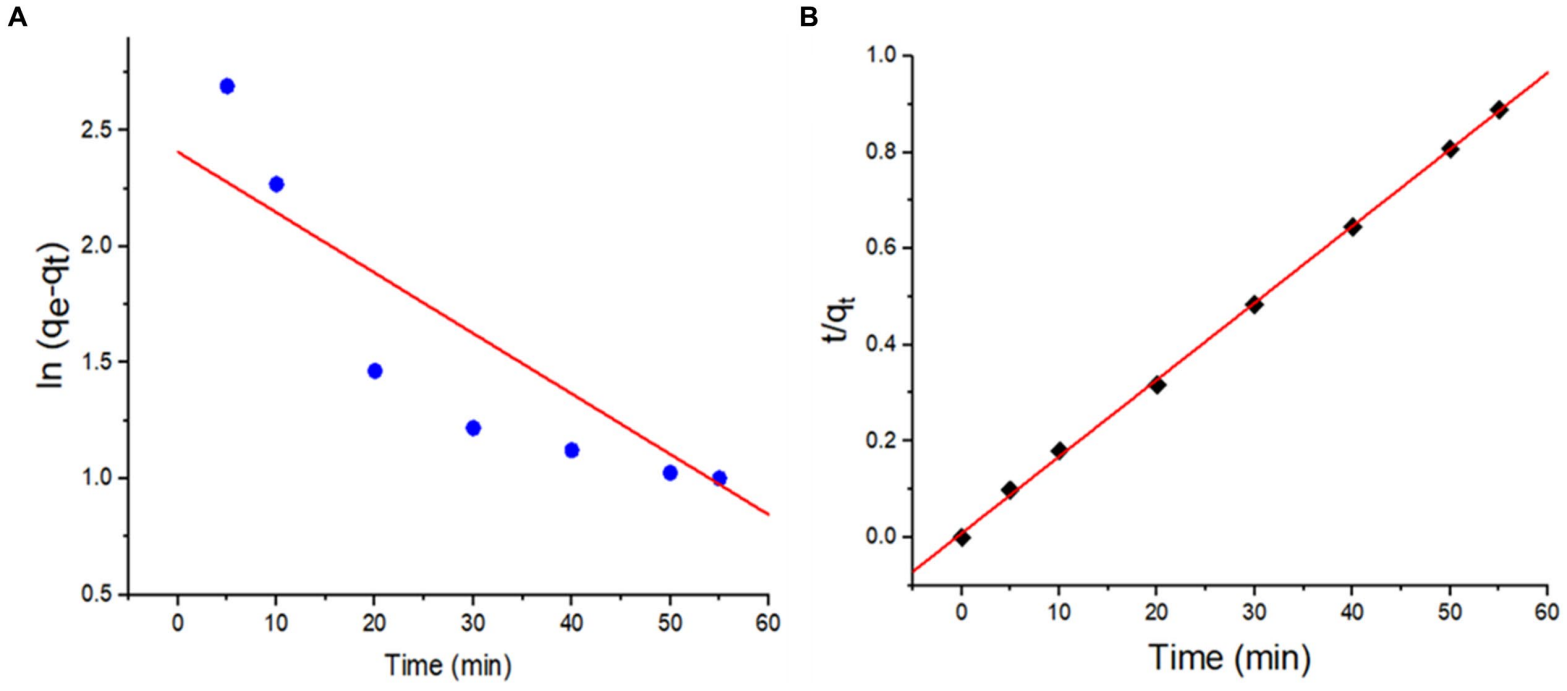
**(A)** pseudo-first-order and **(B)** pseudo-second-order plots of Pb^2+^ biosorption by *B. subtilis* biomass (at pH 6.0, biosorbent dosage 0.3 g, initial Pb^2+^ concentration, 200 mg/L, contact time 60 min; temperature 30°C, 100 mL volume, 100 rpm).

To investigate the mechanism of the biosorption process and the rate of biosorption, the kinetic study of Pb^2+^ ions sorbed by *B. subtilis* biomass was correlated with the pseudo-first-order and pseudo-second-order rate equations. The respective linear fittings of both kinetic models to biosorption equilibrium results were applied, as shown in Figure 13, and the obtained constants and values of the respective kinetic parameters are displayed in Table 4. From Figure 13 and Table 4, the pseudo-second-order kinetic model was best applicable to fit the biosorption data of Pb^2+^ onto *B. subtilis* biomass over the pseudo-first-order kinetic model, according to the higher correlation coefficient constant (R^2^ > 0.999) and the minor variation in the biosorption efficiency between the experimental and calculated values of q_e_. Therefore, the rate-limiting step during Pb^2+^ ion biosorption on the sorbent surface-active sites is highly dependent on the concentration of both sorbent and sorbate in the reaction mixture. Besides, the chemisorption mechanism explained the interaction between the binding of the Pb^2+^ ions and the active biosorbent sites.

### 3.14 Comparison of Pb^2+^ biosorption by *Bacillus subtilis* with other biosorbents

Finally, the Pb^2+^ removal from contaminated water using *B. subtilis* biomass had a superior or comparable performance when compared with other Pb^2+^ biosorbents in terms of contact time, initial metal concentration, and maximum biosorption capacity (mg/g), as clearly proved in Table 5. The obtained biosorption capacity of *B. subtilis* (62.69 mg/g) in the current study was higher than some biosorbent biomass. In contrast, some biosorbent materials, including *B. cereus*, *Arthrobacter* sp., *Lactobacillus brevis*, *Brevibacillus*, and *Bacillus cereus*, showed higher biosorption capacities in the range of 75–150 mg/g. However, the higher initial Pb^2+^ concentration and the lower biosorbent dosage and equilibrium time indicate that *B. subtilis* biomass can be sufficiently used as a biosorbent for Pb^2+^ remediation in contaminated-aqueous systems.

## 4 Conclusion

In the current study, a combination of the DSD and ANN paradigms was performed to optimize the biosorption process of Pb^2+^ using *B. subtilis* biomass. Yeast extract significantly affects biosorption more than other investigated variables in the DSD paradigm. The DSD model displayed a reasonable variation coefficient (R2) value from the ANOVA analysis. However, the lack-of-fit error exhibited significant performance. Therefore, the DSD model was invalid for optimizing the Pb^2+^ sorption process. In contrast, the ANN model exhibited lower error values when compared with the DSD paradigm. The forecasting capability of the ANN model proved satisfactory for predicting the Pb^2+^ biosorption process. The equilibrium data conformed better to Langmuir’s isotherm and the pseudo-second-order model, suggesting a monolayer sorption and chemisorption mechanism. Therefore, it can be concluded that a successful prediction of Pb^2+^ biosorption using *B. subtilis* biomass was obtained through the artificial intelligence paradigm, hinting at their promising application in optimizing the removal of Pb^2+^ ions from wastewater systems.

Nevertheless, the successful application of artificial intelligence in the biosorption process requires further studies using various heavy metal ions. Additional research is required to compare the efficiency of dead and live *B. subtilis* biosorbents for heavy metal removal from contaminated water. A major challenge to be addressed is determining the economic feasibility of large-scale applications, particularly in developing countries.

## Data availability statement

The original contributions presented in the study are included in the article/Supplementary material, further inquiries can be directed to the corresponding authors.

## Author contributions

RE-S: Conceptualization, Investigation, Methodology, Software, Writing – original draft, Writing – review & editing. MK: Investigation, Methodology, Software, Writing – review & editing. MHA: Investigation, Methodology, Software, Writing – original draft, Writing – review & editing. MEZ: Investigation, Methodology, Writing – review & editing. AE: Investigation, Methodology, Writing – original draft, Writing – review & editing.

## Footnotes

^1^ http://www.ncbi.nlm.nih.gov/Blast
