# Supplementary material for: Innovative optimization for enhancing Pb2+ biosorption from aqueous solutions using Bacillus subtilis
Source: Front Microbiol. 2024 Aug 8;15:1384639. doi: 10.3389/fmicb.2024.1384639 (PMC11338800; doi:10.3389/fmicb.2024.1384639)
Supplement: Supplementary file 1 [file Table_1.pdf]

Innovative optimization for enhancing Pb^{2+} biosorption from aqueous solutions using *Bacillus subtilis*

Reyad M. El-Sharkawy[1*], Mohamed Khairy[2,3], Mohamed H. H. Abbas[4], Magdi E. A. Zaki[2], Abdalla E. El-Hadary[5]

[1]Botany and Microbiology Department, Faculty of Science, Benha University, Benha 13511, Egypt; r.m.elsharkawy@fsc.bu.edu.eg

[2]Chemistry Department, College of Science, Imam Mohammad Ibn Saud Islamic University (IMSIU), Riyadh 11623, Saudi arabia; mkomran@imamu.edu.sa; mezaki@imamu.edu.sa

[3]Chemistry Department, Faculty of Science, Benha University, Benha 13518, Egypt

[4]Soils and Water Department, Faculty of Agriculture, Benha University, Benha, Egypt

[5]Biochemistry Department, Faculty of Agriculture, Benha University, Benha, Egypt

*** Correspondence:**
Reyad M. El-Sharkawy
r.m.elsharkawy@fsc.bu.edu.eg, https://orcid.org/0000-0003-1319-9066

Figure S1: Graphs of three-dimensional surface plots of SDS for the Pb^{2+} removal by *B. subtilis*, illustrating the interactive configuration of each two set of the independent variables, keeping the other variables at the middle level.

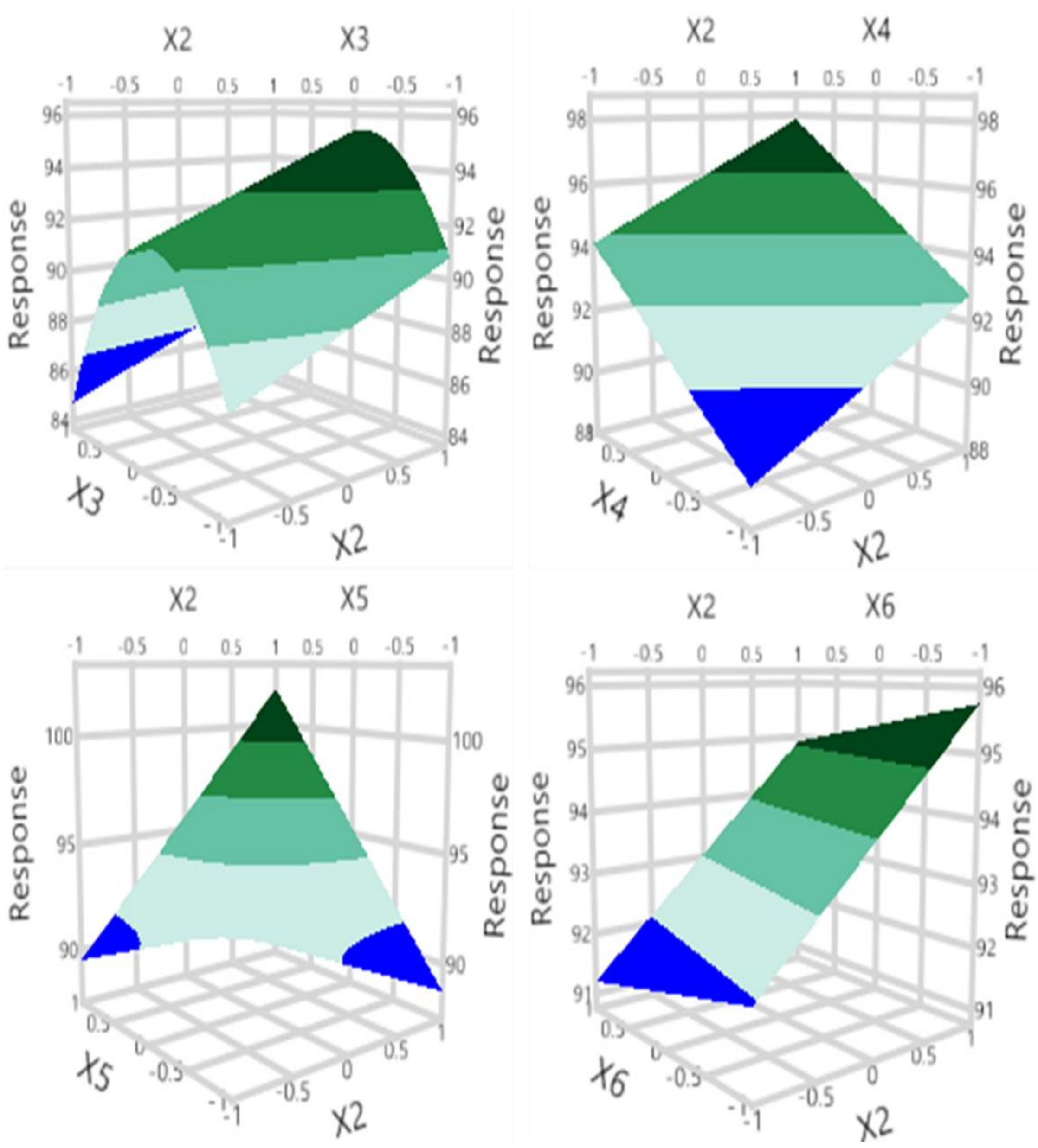

A) interaction of temperature (X_2, °C) with the other variables.

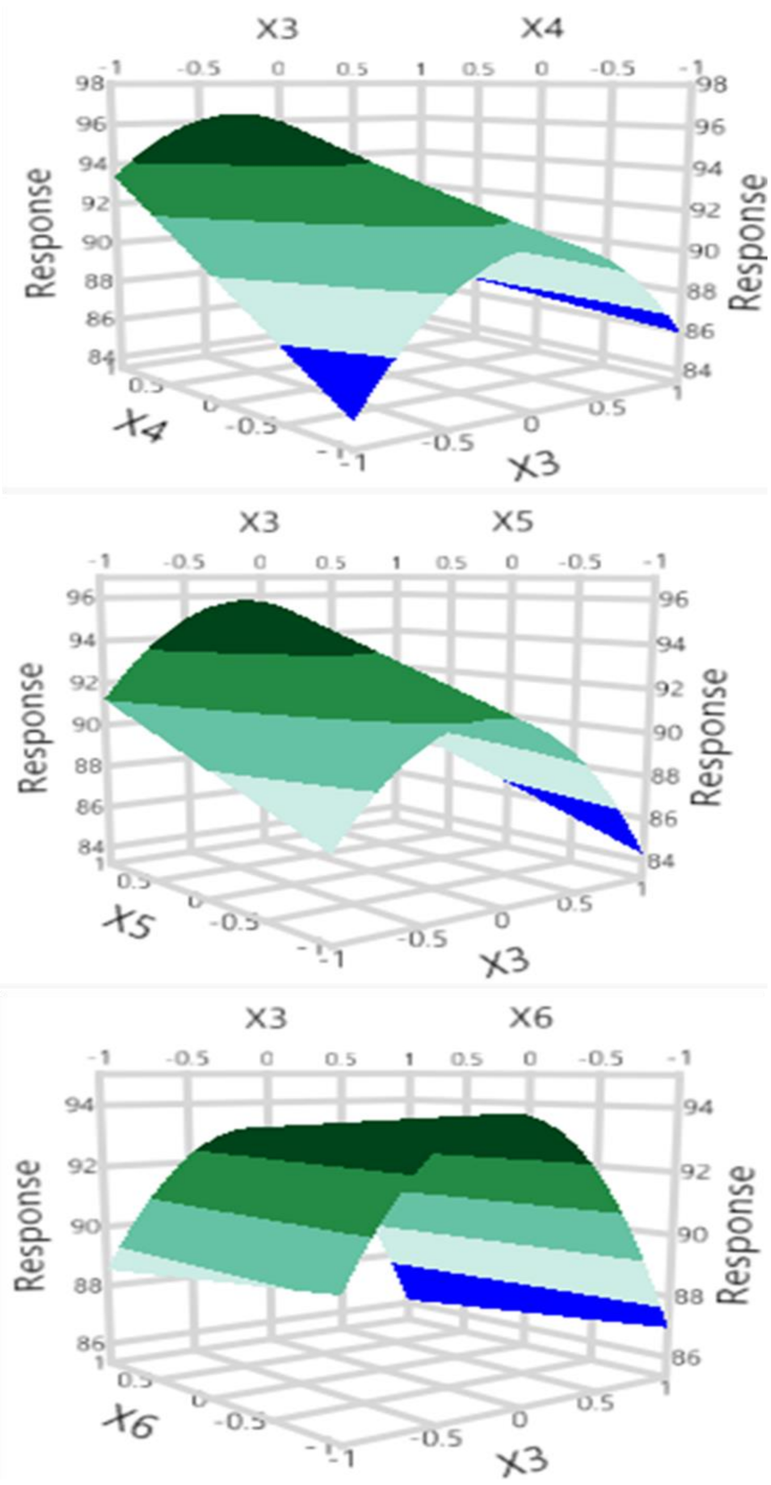

B) interaction of glucose (X_3, %) with the other variables.

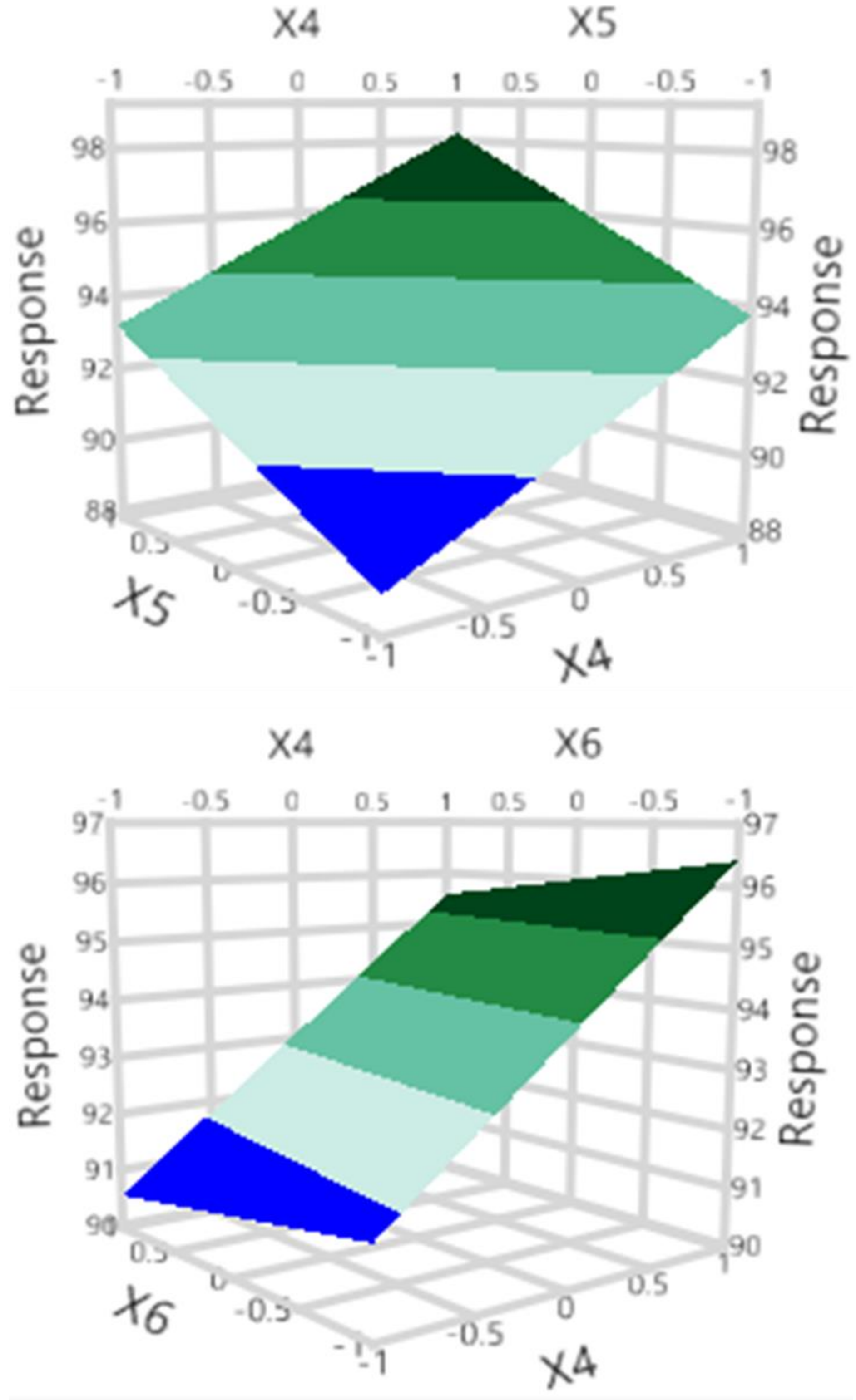

C) interaction of yeast extract (X_4, %) with the other variables.

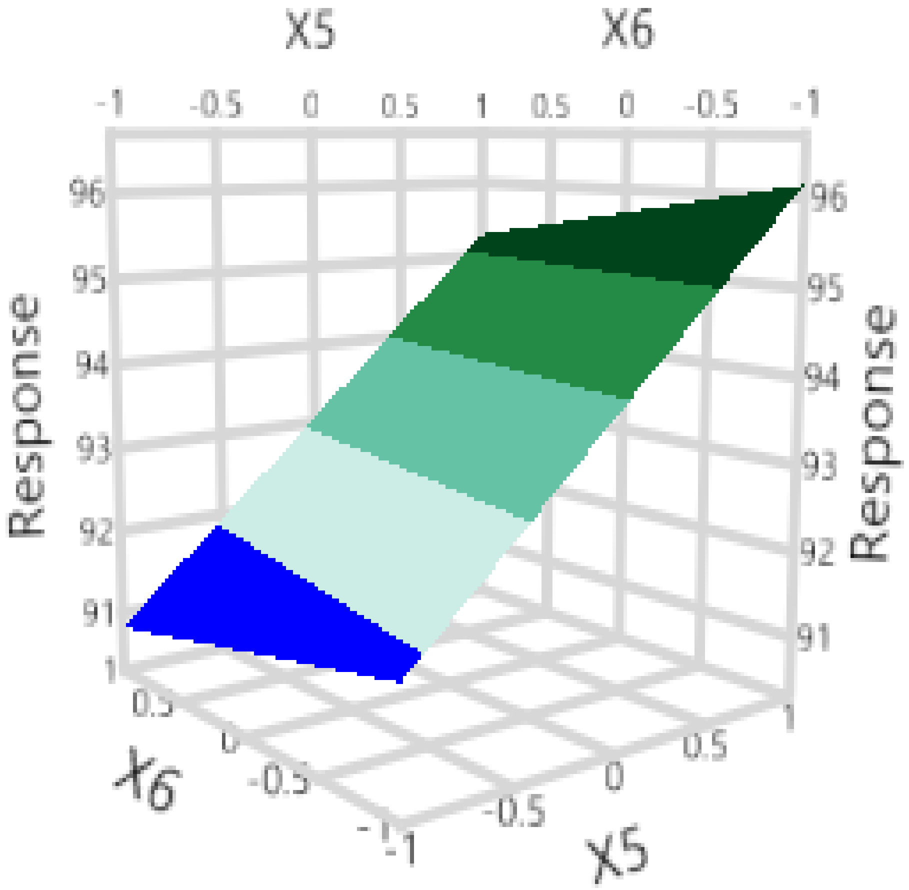

D) interaction of $MgSO_4.7H_2O$ (%) (X_5, %) with K_2HPO_4 (X_6, %) variables.

Figure S2: Graphs of three-dimensional surface plots of ANN for the Pb^{2+} removal by *B. subtilis*, illustrating the interactive configuration of each two set of the independent variables, keeping the other variables at the middle level.

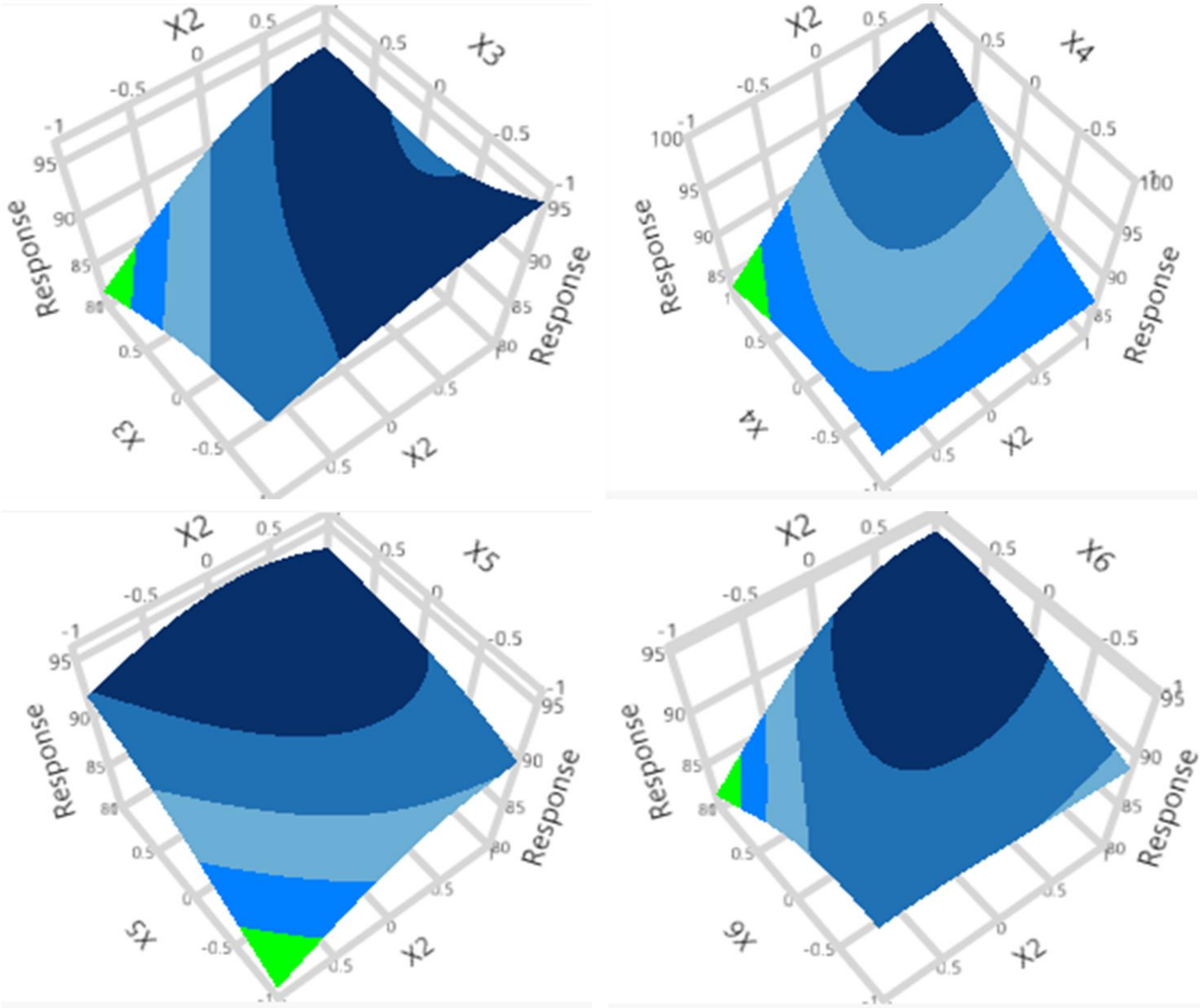

A) interaction of temperature (X_2, °C) with the other variables.

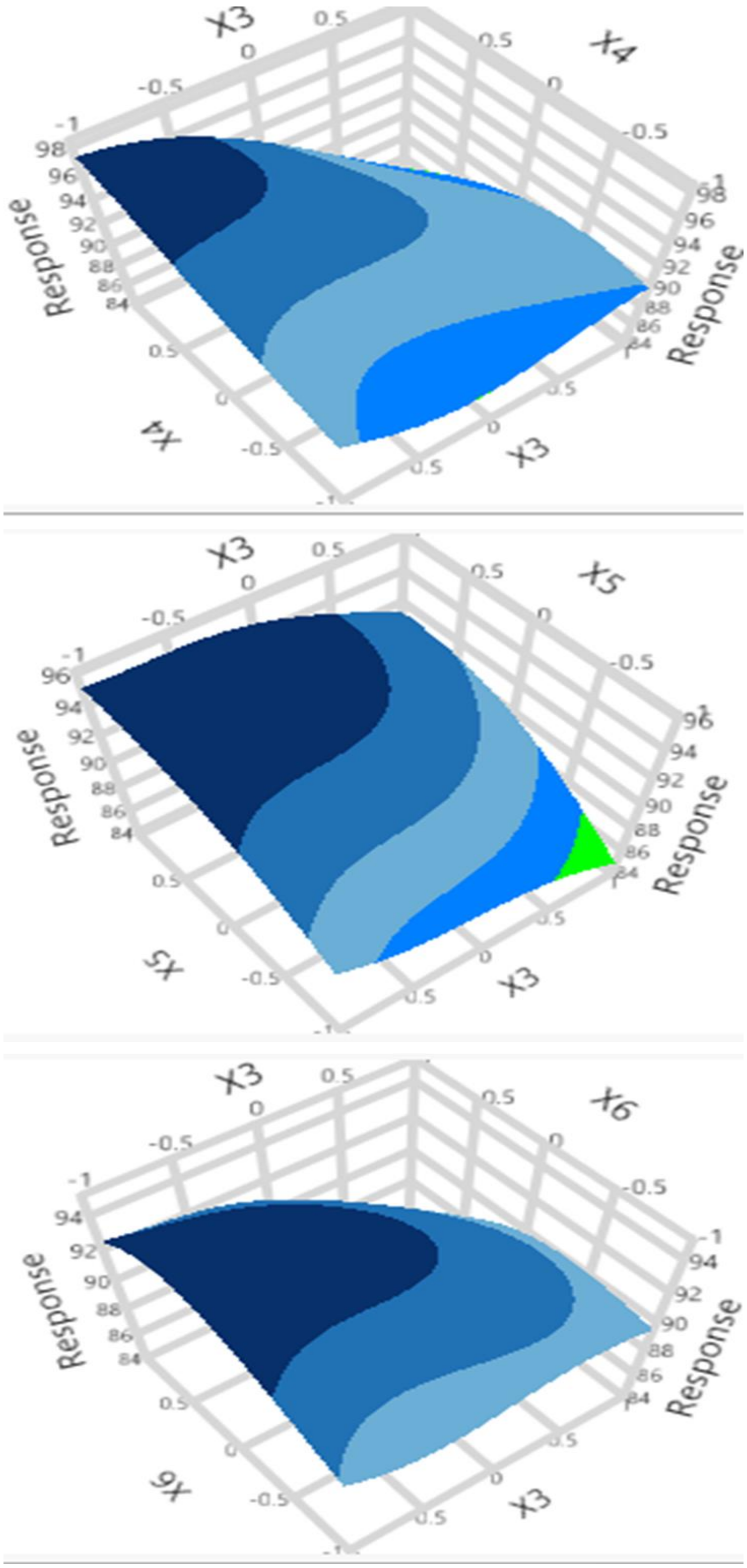

B) interaction of glucose (X_3, %) with the other variables.

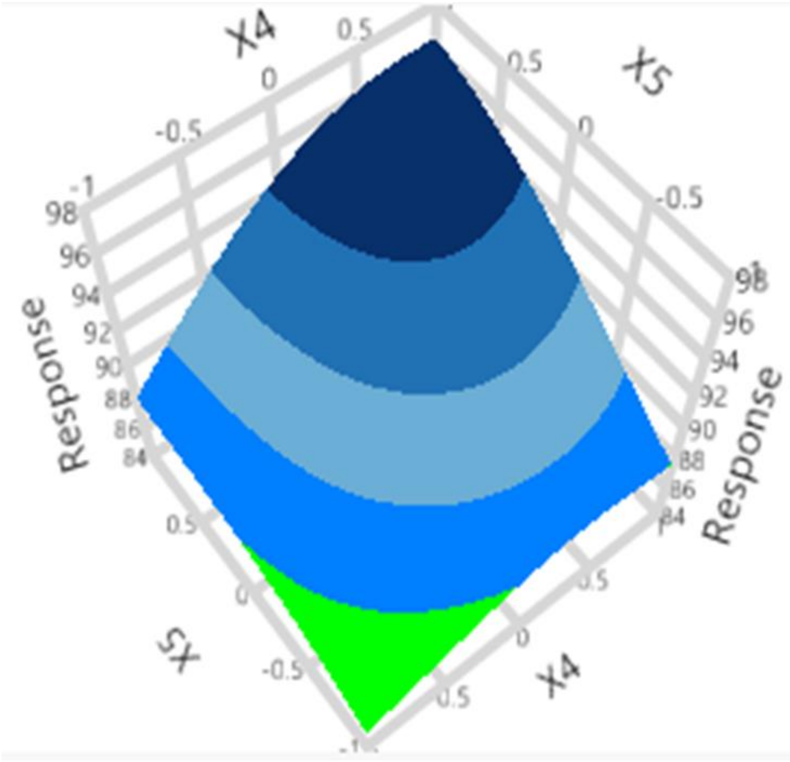

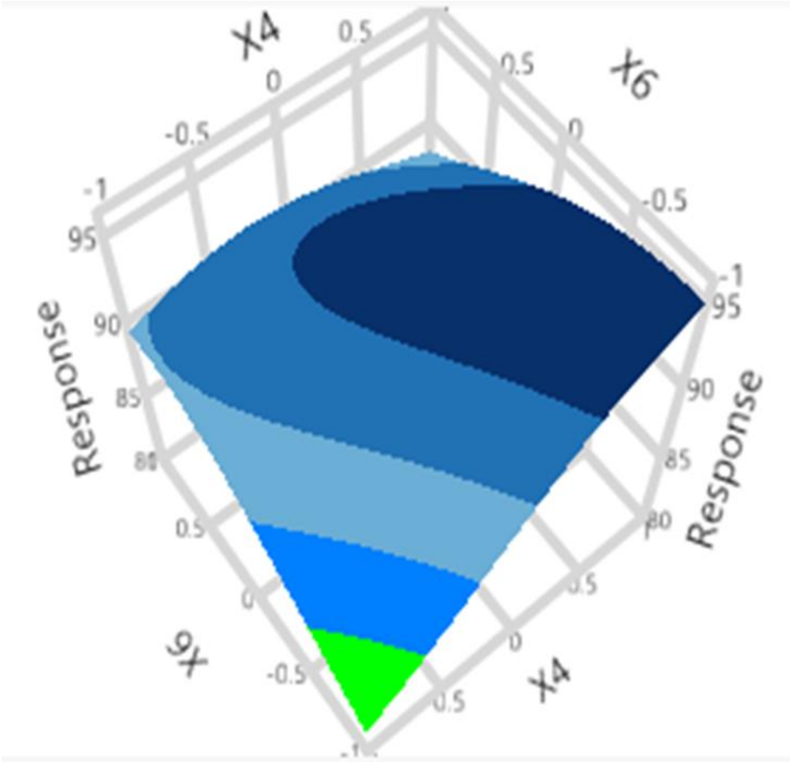

C) interaction of yeast extract (X_4, %) with the other variables.

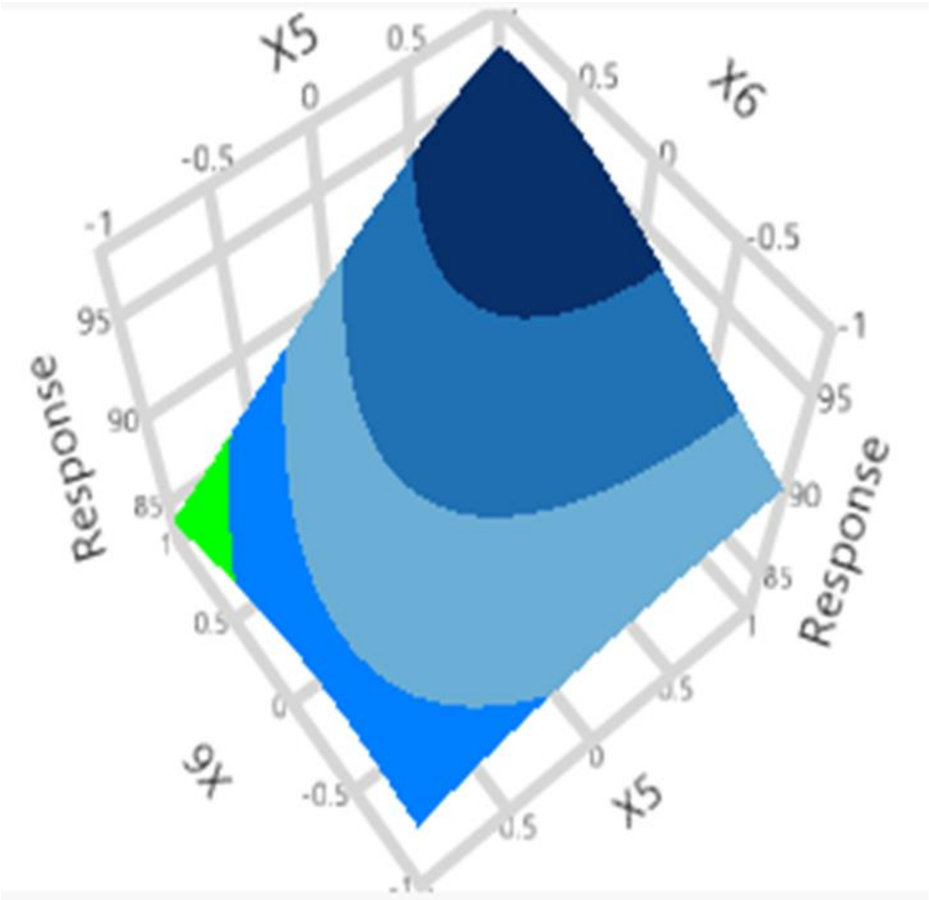

D) interaction of $MgSO_4.7H_2O$ (%) (X_5, %) with K_2HPO_4 (X_6,%) variables.

Table S1: Comparison of the DSD and ANN models statistics for the Pb^{2+} removal by *B. subtilis*. Variation coefficient (R^2), root mean squared error (RMSE), average absolute deviation (AAD), and sum of error's squares (SES) for each operated model were detected.

		**Model**	
		**DSD**	**ANN**
Training	R^2	0.8345	0.9865
	RMSE	2.622	0.7496
	Frequency	11	11
Validation	R^2	0.8858	0.9964
	RMSE	2.539	0.4482
	Frequency	6	6
Overall model	R^2	0.9828	0.9999
	RMSE	1.3925	0.0468
	SES	634.706	88.789
	AAD	1.5077	0.0016
	Frequency	17	17

Table S2: The predicted and experimental values of the Pb^{2+} removal by *B. subtilis* under optimal conditions.

**Independent variable**	**Optimum conditions**	**Experimental conditions**
pH	6.1	6.1
temperature (°C)	30.8	30
yeast extract (%)	1.7	1.7
Glucose (%)	1.5	1.5
$MgSO_4.7H_2O$ (%)	0.2	0.2
K_2HPO_4 (%)	0.2	0.2
Pb^{2+} removal (%)	96.98	96.12
